# Stability analysis of rock slope and calculation of rock lateral pressure in foundation pit with structural plane and cave development

**DOI:** 10.1038/s41598-022-12765-6

**Published:** 2022-05-24

**Authors:** Jin Xu, Yansen Wang, Changchun Li

**Affiliations:** State Key Laboratory for Geomechanics and Deep Underground Engineering, China University of Mining and Technology, Xuzhou, 221116 Jiangsu China

**Keywords:** Civil engineering, Applied mathematics

## Abstract

In this study, numerical simulations were carried out to analyze the influence of caves in different positions and shapes, in combination with structural planes, on the stability of the slope and the failure characteristics of a rock slope in a deep foundation pit with high inclination structural planes and cave development. The schemes for substituting a single karst cave for karst caves were constructed. Based on the penetration failure characteristics of karst caves between parallel structural planes, calculation methods for the safety factor of the rock foundation pits and the upper bound of the lateral pressure of supporting structures under the combined influence of the caves and structural planes have been developed, which can be used to assess the safety factor of a rock mass and to calculate the lateral pressure under complex geological conditions. The example of Xuzhou Metro Line 3 Sanhuan South Road Station is taken as an example to verify. The displacement calculation results show that the proposed rock and soil pressure calculation method is close to the actual measured value.

## Introduction

A variety of methods have been developed to study the stability of soil slopes in foundation pits. However, for rock slopes, the current consensus is that the failure to rock slopes is related to the structure of the rock mass or the structural plane^1^, but the mechanism of failure has not yet been fully determined. Current theoretical methods for analyzing the stability of pit slopes are mainly limit equilibrium method^2–5^, limit analysis method^6–9^, numerical analysis^10–12^, fuzzy analysis^13–15^, and reliability analysis^16–18^. Few studies have been conducted on the stability of rock slopes in areas with well-developed karst caves. This is primarily due to the difficulties in converting between numerical models and theoretical analyses of karst caves.

Existing numerical models used to analyze the influence of karst caves on the stability of foundation pits are generally stochastic geological models established using the Monte Carlo method^19^. These methods are only applicable to certain areas and are heavily dependent on geological exploration data. The analysis results are only applicable to certain engineering conditions and thus have great limitations^20^. The theoretical analysis of karst caves, on the other hand, is generally made on the basis of consideration of the overall stability with a discounted safety factor based on engineering experience, and does not analyze the influence of factors such as the position and shape of the karst cave on the final fracture surface of the pit slope. For caves in special positions, the positional relationship to the structural plane may change the original failure path along the structural plane^21^. Therefore, uniform discounting of rock lateral pressure or increasing the stability safety factor for design is not compatible with reality.

As early as 2001, scholars have done a lot of indoor tests on the fracture penetration mode of rock masses, and concluded that fractures form penetration by branching fracture inflection zigzagging under static load^22–25^. On the basis of previous research, Yang^26^ carried out uniaxial compression test on discontinuous three-fissure sandstone and concluded that the destabilization failure of discontinuous three-fissure sandstone sample was caused by many crack propagation and confluence from the crack tip. Huang^27^ established the dynamic process of rock high slope development under unloading conditions. According to the laboratory similar model test, it is considered that the influence range of tensile stress gradually decreases with the increase of inclination of rock bridge. Chen^28^ carried out uniaxial compression tests on the open rock bridge, and concluded that the main crack of the rock bridge was inflected from the lower crack tip to the upper part to penetrate the upper end face, and multi-stage damage occurred in the process of penetration. The above research results are generally based on the conclusions summarized by indoor tests, and the theoretical calculation model of the penetration damage mode has not been realized, so it cannot be used in engineering practice.

In this paper, numerical simulation method is used to analyze the mode of single karst cave in different shapes and locations affecting the final failure of rock slope, and the formation failure path of karst cave groups in the slope, so as to find a scheme of replacing karst cave groups with single karst cave. According to the above research results, a formula for calculating stability of rock slope is put forward based on the principle of upper limit analysis. Through this formula, the relationship between safety factor of slope and mechanical parameters of rock mass and structural plane is further analyzed, and a method for calculating the position and shape of karst cave which is most disadvantageous to stability of foundation pit and the residual sliding force (earth pressure) of rock slope is provided for engineers.

## Failure modes of rock slopes containing differently shaped karst caves

A 2-D plane strain model was used to simulate the final failure range and mode of the rock mass under different cave shapes and position conditions.

**Figure 1:**
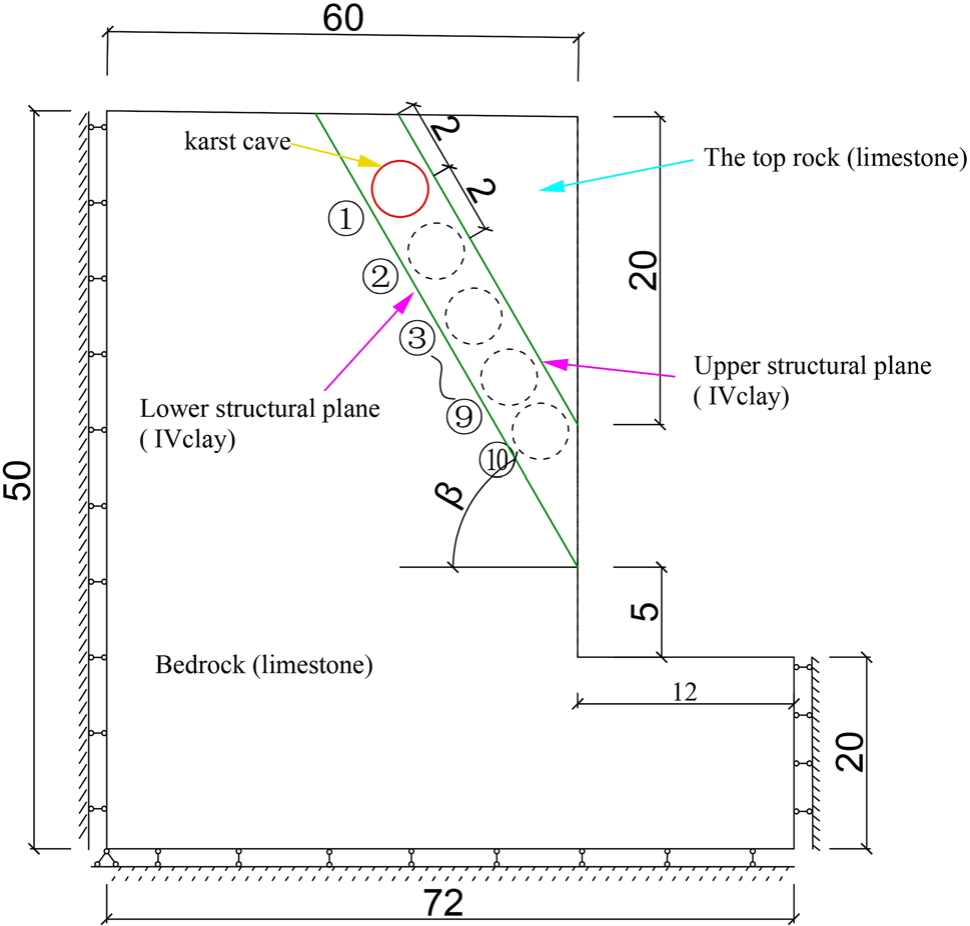
Numerical model of the foundation pit (unit: m).

The foundation pit model is shown in Fig. 1, the ideal elastoplastic constitutive equation obeying the Mohr–Coulomb criterion and the non-associated flow rule was used for the model. The parameters of the rock mass and structural planes are presented in Tables 1 and 2.

**Table 1:** Rock mass parameters of the calculation model.

Name	Density (kg/m^3^)	Young's modulus (Pa)	Poisson's ratio	Cohesion (Pa)	Internal friction angle (°)	Compressive strength (Pa)
Limestone	2300	21.0e9	0.22	3.0e6	30	1.0e6

**Table 2:** Structural plane parameters of the calculation model.

Name	Normal stiffness (Pa)	Shear stiffness (Pa)	Cohesion (Pa)	Internal friction angle (°)	Compressive strength (Pa)
Structural plane (IV clay)	2.0e9	1.0e9	2.0e5	15	1.0e5

In Tables 1 and 2, limestone was the object of analysis and the middle values of the empirical ranges of the mechanical parameters for moderately weathered limestone were taken as the model parameters by referring to the Engineering Geology Handbook (5th edition). In order to ensure the generality of model parameter selection, the failure mode of rock mass under combined action of karst cave and structural plane is analyzed by taking the middle value of empirical parameter range of limestone as an example. The determination of failure form of rock mass and calculation of safety factor will be further analyzed in the theoretical part of Chapter 4. Therefore, the parameters of the numerical model do not affect the generalization of the research conclusions.

The parameters of the main slip fracture surface were determined based on fully weathered type IV clay. The inclination angle β of the slope’s structural plane ranged from 60° to 80°. Starting from the upper structural plane and extending towards the bottom of the foundation pit, a cave calculation point was set up at 2 m intervals, and the failure modes were recorded for 10 location points for each working conditions. The apparent distance between the parallel structural planes and the excavation surface was 5 m, and the true distance varied with the inclination of the structural plane in Fig. 1. Based on the center line of the foundation pit width, a half side model of rock foundation pit is established, which is 5 times of the excavation width of the foundation pit. The left and right boundaries of the model are constrained by the displacement in the X direction (horizontal direction), and the bottom of the model is constrained by the displacement in the Z direction (vertical direction).

In order to facilitate the description of the influence of the karst cave’s shape on the foundation pit failure mode, the diameter-to-distance ratio (DDR) was introduced, which is defined as the ratio of the diameter of a circular cave (or the major axis of an elliptical cave) to the true distance between the structural planes. The range of values for the distance to diameter ratio is 0.9–0.2.

The criterion for stability and failure of foundation pit slope has the following points in the analysis of soil slopes: first, the model calculation does not converge^29,30^; second, plastic penetration zone appears in rock mass^31–35^; third, the rock mass deformation reaches the preset value^36,37^. Plastic zone penetration is usually used to determine the stability of foundation pit in the above three methods. The calculation of non-convergence requires artificial setting of calculation unbalance point, and the maximum deformation value needs to be preset when rock deformation reaches the preset value. For complex rock slopes, the two methods lack in-situ measured analysis, and even the calculation results may show that the rock mass has not reached the failure state. Therefore, there are some disadvantages in analyzing the failure form of complex rock slope. However, it is a common method to analyze the failure state of slope according to the fracture penetration area of rock mass, which has achieved good results in the analysis of soil slope.

In this paper, the following two methods are used to identify rock foundation pit by referring to the method of stability discrimination of soil slope: first, the upper rock mass slides along a structural plane and realizes a straight line between the bottom of the pit and the top of the pit, resulting in dislocation between the upper rock mass and the bedrock. Secondly, the existence of karst caves between structural planes leads to the penetration between structural planes, forming broken-line fracture and realizing the broken-line penetration between pit bottom and pit top.

### Influence of the inclination angle of the structural plane on the failure mode of the rock slope

**Figure 2:**
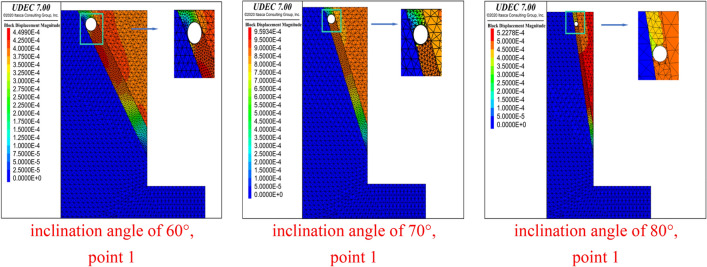
Displacement of the starting point of the penetration failure under different inclination angles of the structural plane (DDR = 0.9).

Simulations were carried out for working conditions with inclination angles of 60°, 70° and 80° respectively, and the displacement map at point 1 for each inclination angle are shown in Fig. 2.

With a DDR of 0.9, for example, the cave penetration failure occurred from point 1 at the top of the foundation pit under all three conditions. As the angle of inclination of the structural plane increases, the angle between the crack and the lower structural plane also gradually expands, that is, the failure mode changed from shear failure to tensile failure.

**Figure 3:**
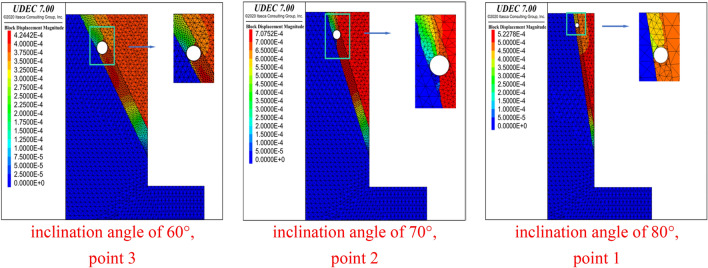
Displacement of the starting point of the penetration failure under different inclination angles of the structural plane (DDR = 0.9).

Figure 3 shows the end point for outward penetration failure to the surface of the structure at different inclination angles, i.e. no penetration failure (to the outside of the foundation pit) occurs in the area below this point. For an inclination angle of 60°, the penetration failure extends to point 3; while for the 70° dip case, it extends to point 2 and for the 80° dip case, the penetration failure only occurs at point 1.

**Figure 4:**
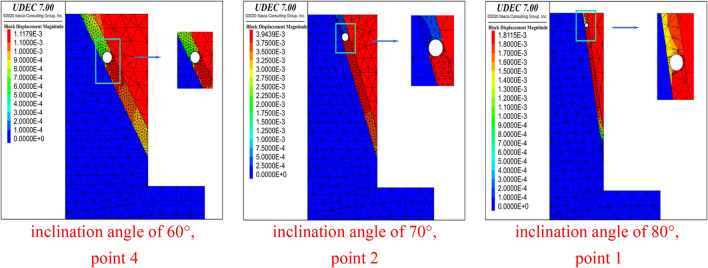
Displacement of the starting point of the penetration failure under different inclination angles of the structural plane (DDR = 0.8).

When the DDR was decreased 0.8 (Fig. 4), the starting point of the penetration failure in the circular cave did not change, yet the end point of the penetration failure changed from point 3 (DDR = 0.9) to the fourth point (DDR = 0.8) for an inclination angle of 60°. However, for inclination angles of 70° and 80°, the end point did not change. The failure of the rock bridge changed from shear failure to tensile failure. It can be concluded that the penetration failure mode of karst caves is related to the size of the rock bridge between the karst cave and the structural plane and the inclination of the structural plane.

### Influence of the shape of a karst cave between parallel structural planes on the failure mode

**Figure 5:**
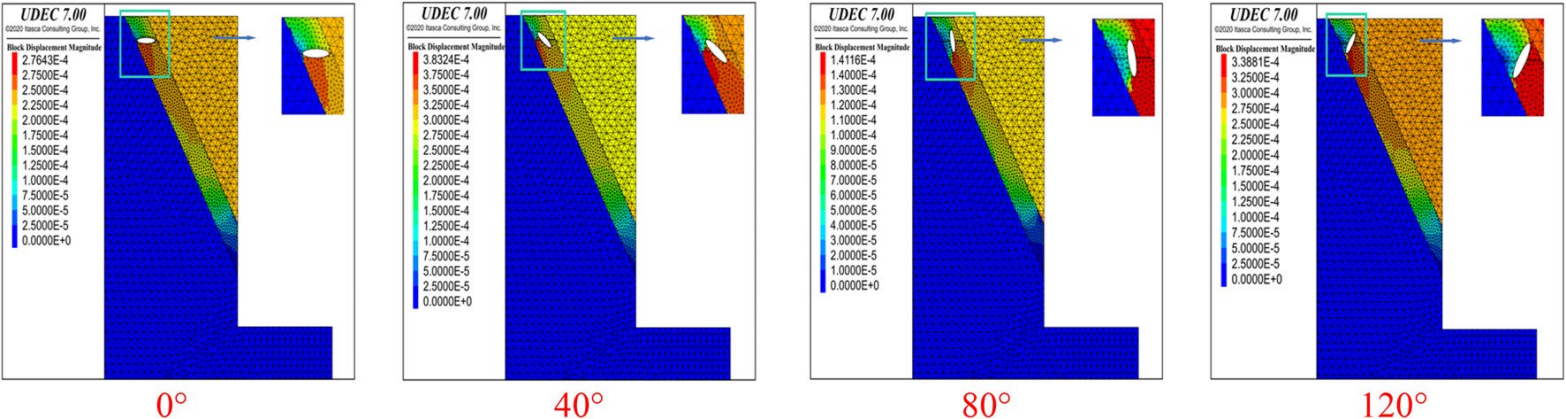
Failure modes of elliptical caves with different angles at point 1 for an inclination angle of 60° (DDR = 0.9).

In order to analyze the influence of the shape of the cave on the failure mode of a rock mass between structural planes, circular caves were replaced by elliptical caves under the same DDR and the cave long-axis angles of 0°, 40°, 80° and 120° (rotated clockwise with the horizontal axis as the reference) were taken for numerical analysis of the same parameters, with their results shown in Fig. 5.

When the major axis of the cave was horizontal (0°), the penetration failure extended along the two ends of the major axis to the parallel structural planes, with tensile failure occurring in the upper rock bridge and shear failure in the lower rock bridge. After a 40° turn, the start of the cave penetration began to extend from the top of the foundation pit towards the position of the karst cave, and the upper rock bridge changed from tensile to shear failure. After rotating at an angle of 80°, the penetration failure extended towards the upper and lower structural planes along the major axis. And the two cracks were approximately parallel, with shear failure occurring in both of them. the long axis was rotated to an angle of 120°, its penetration points unfolded along the ends of the long axis towards the parallel structural planes, and the failure mode of the rock bridge appeared to be tensile failure.

**Figure 6.**
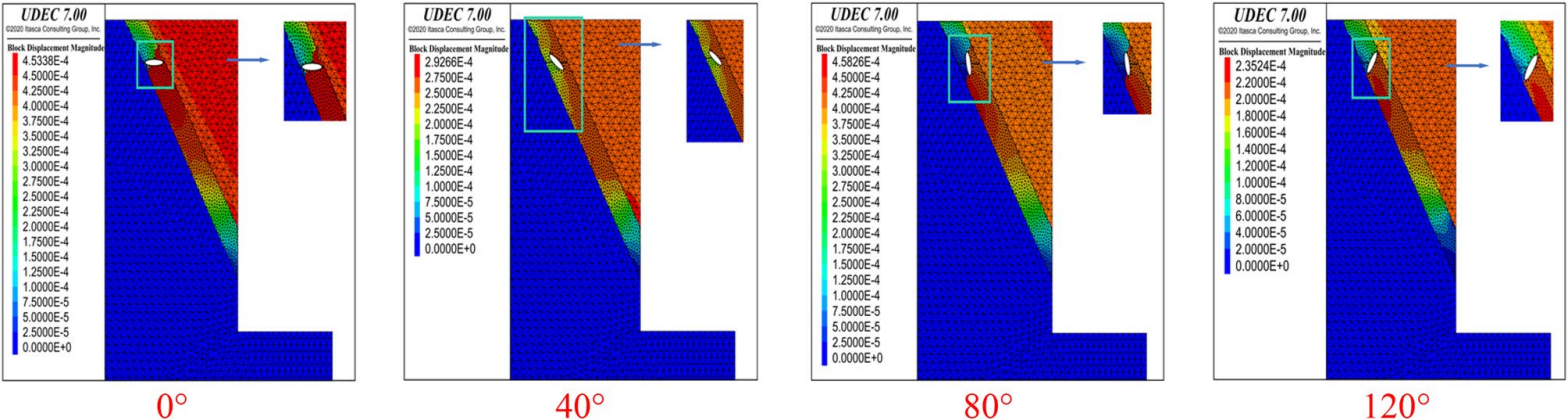
Failure modes of elliptical caves with different angles at point 2 for an inclination angle of 60° (DDR = 0.9)

As shown in Fig. 6, for the structural plane with an inclination angle of 60°, the failure of the cave with a horizontal major axis (0°) penetrated the upper and lower structural planes at the second calculation point, exhibiting overall shear failure. When the major axis angle increased to 40°, the cave and the surrounding rock mass underwent integral slumping failure. When the major axis of the cave was rotated to 80°, the failure mode was similar to that of 0°, i.e., shear failure passing through the cave. When the angle was further increased to 120°, the failure mode was the same as that of the first calculation point. Due to space limitations, the conditions at other inclination angles will not be discussed here. The failure at the 10 calculation points was analyzed by changing the shape of the cave, the DDR and the angle of the major axis, and the conclusions that follow were obtained.

The analysis of each working condition will not be repeated here for lack of space. The analysis of calculation points 1–10, by varying the cavity shape, DDR and major axis angle, leads to the following conclusions.

**Figure 7:**
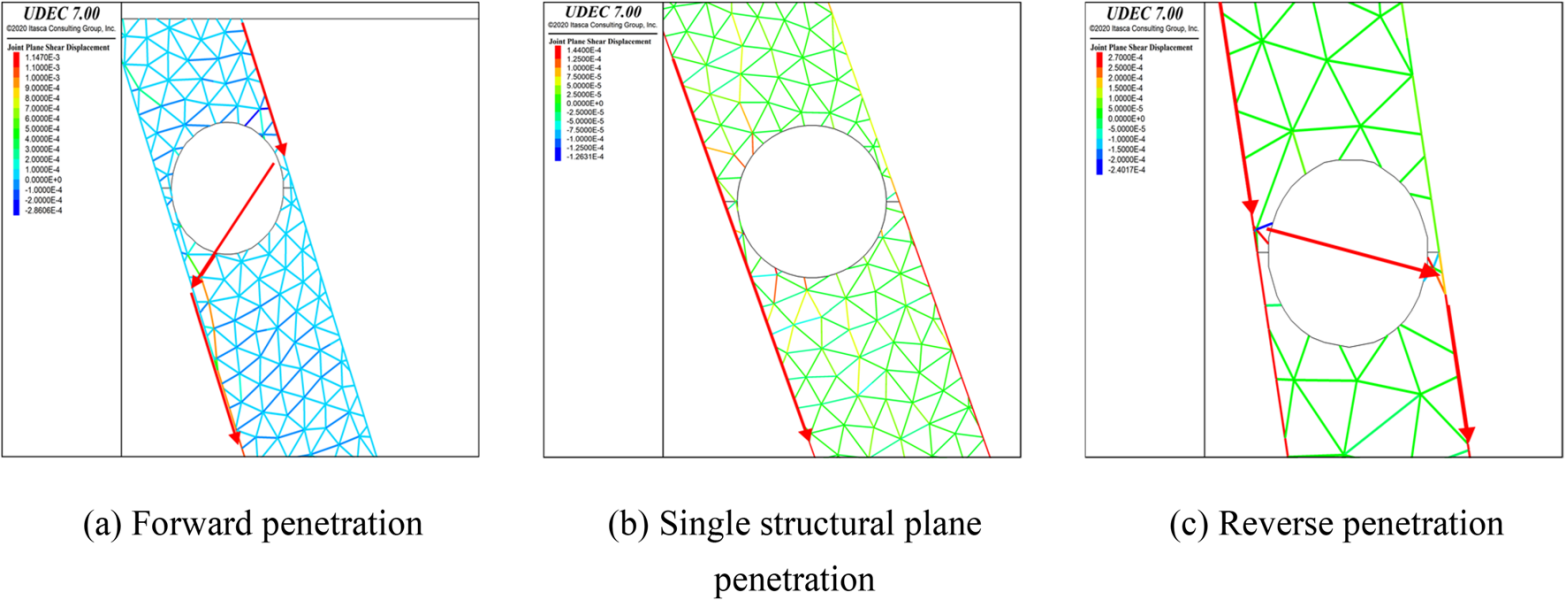
Three failure modes of rock slope with structural plane and karst cave.

1. For inclination angles of 60°–80°, the presence of karst caves may cause penetration failure of the structural plane. However, there is a minimum DDR value for the occurrence of penetration failure at each inclination angle. When the DDR is less than the minimum value, overall slip failure occurs.
2. For a given DDR value, as the dip angle of the structural surface increases, the position where the circular cave penetration occurs gradually moves towards the top of the foundation pit, that is, the area where the overall failure of the rock mass occurs expands.
3. Regarding the influence of the major axis angle of the elliptical cave on the occurrence of penetration failure, when the inclination angle of the structural plane is 60° and 70°, and the major axis angle is 0°–40° and 80°–120°, the cave underwent penetration failure. When the major axis angle is 40°–80°, overall slip failure occurs.
4. For rock slopes developed by combination of structural plane and karst cave, their failure modes are divided into three types as shown in the Fig. 7a–c: forward penetration (Slip body slides along upper structural plane at the shallow part of foundation pit, and the whole slides along lower structural plane after the crack penetrates the karst cave towards the outer side of foundation pit.), single structural plane penetration and reverse penetration (The sliding body slides along the lower structural plane near the free surface of the foundation pit. After the crack penetrates the karst cave towards the inner side of the foundation pit, the whole slides along the upper structural plane.).

## Penetration paths of karst cave groups based on single cave failure mode

In addition to their individual presence, caves in foundation pit slopes are more often found in groups. In order to simplify the theoretical analysis of karst cave groups, the feasibility of substituting groups of karst cave groups is analyzed here in order to achieve replacement of the same failure path.

**Figure 8:**
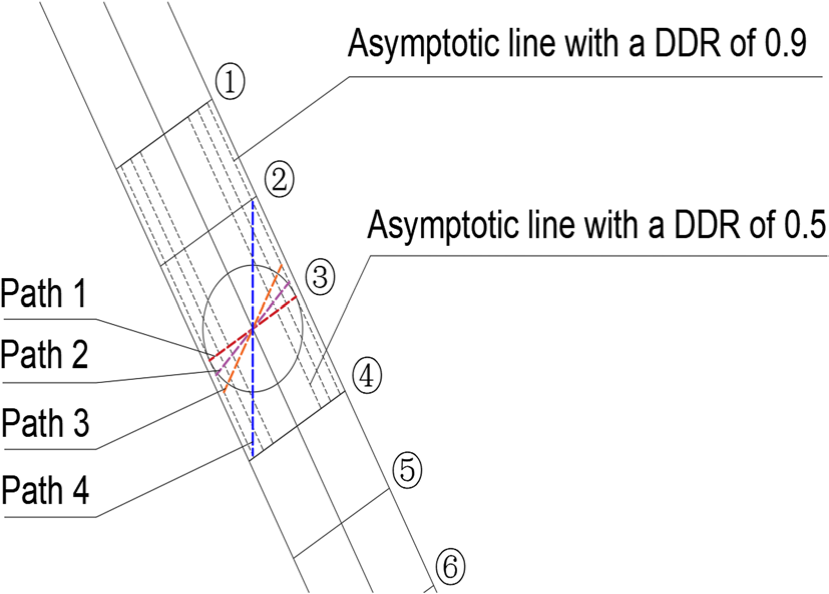
Similar failure modes of karst cave groups and circular karst cave.

Taking the 60° structural face inclination as an example (Fig. 8), an asymptotic line with a DDR of 0.9 was drawn parallel to the structural plane. Then, four possible failure paths were selected based on possible failure angle of the rock mass. Path 1 was perpendicular to the line and 30° from the horizontal plane, path 2 was 45° from the horizontal plane, path 3 was 60° from the horizontal plane (30° to the internal friction angle of the rock mas), and path 4 was perpendicular to the horizontal plane.

**Figure 9:**
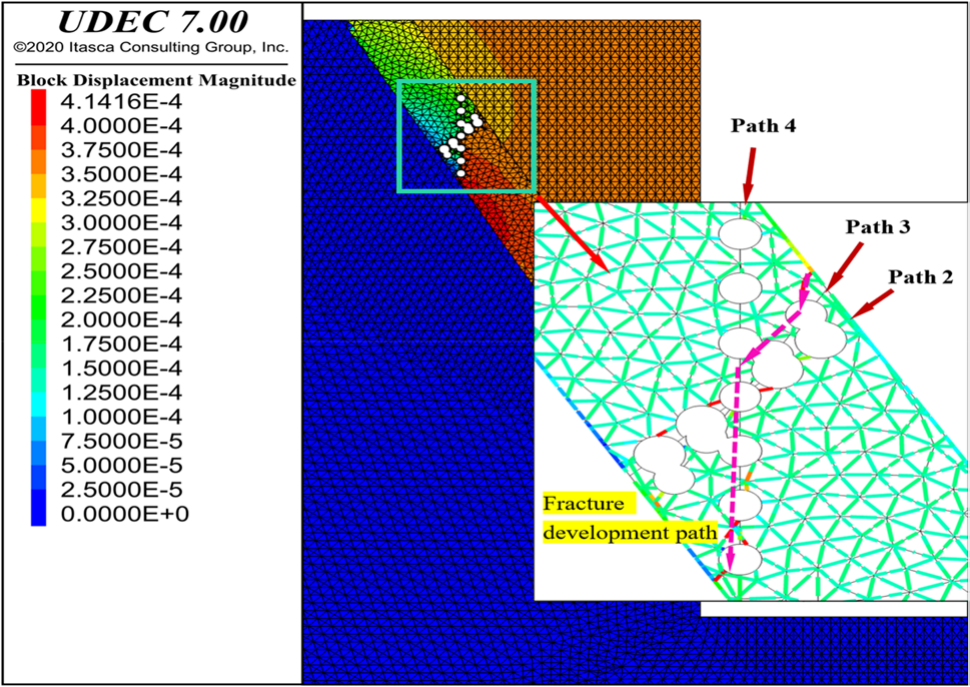
Penetration failure paths of karst cave groups.

The analysis used the DDR of individual caves as a reference to select the specified path, and by varying the total DDR of the cave group, the parameters of the cave group that need to achieve a similar failure path and the minimum total DDR required for the cave group were determined. For simplification, the distance between each karst cave was the same, and four working conditions were simulated (i.e., 3, 5, 7, and 9 karst caves). The cloud diagram and the substitution scheme are shown in Fig. 9 and Table 3, respectively.

**Table 3:** Karst cave group substitution table.

Parameters of single cave					Parameters of cave groups					
No	Failure path	Radius/major semi-axis(mm)	DDR	Range of major and minor axis ratio	Radius (mm)	Cave distance (mm)	Analysis path	Number of caves	Total DDR	Failure mode
1	1 1	1125 1000	0.9 0.8	3–9 3–5	250	875	1	3	0.6	Overall
2					292	833	1	3	0.7	Penetration
3					200	463	1	5	0.8	Overall
4					225	450	1	5	0.9	Penetration
5					143	327	1	7	0.8	Overall
6					161	321	1	7	0.9	Overall
7					111	254	1	9	0.8	Overall
8					125	250	1	9	0.9	Overall
9	2 2	1165 1035	0.9 0.8	≥ 7 ≥ 8	216	942	2	3	0.5	Overall
10					259	897	2	3	0.6	Penetration
11					181	489	2	5	0.7	Overall
12					207	475	2	5	0.8	Penetration
13					111	350	2	7	0.6	Overall
14					129	344	2	7	0.7	Penetration
15					115	262	2	9	0.8	Overall
16	3 3 3 3	1125 1000 1155 1300	0.9 0.8 0.8 0.9	1 1 ≥ 7 ≥ 7	241	1024	3	3	0.5	Overall
17					289	968	3	3	0.6	Penetration
18					144	567	3	5	0.5	Overall
19					173	551	3	5	0.6	Penetration
20					124	386	3	7	0.6	Overall
21					144	378	3	7	0.7	Penetration
22					128	288	3	9	0.8	Overall
23	4 4	2250 2000	0.9 0.8	≥ 10 ≥ 9	250	1750	4	3	0.3	Overall
24					333	1583	4	3	0.4	Penetration
25					200	925	4	5	0.4	Overall
25					250	875	4	5	0.5	Penetration
26					143	655	4	7	0.4	Overall
27					179	631	4	7	0.5	Penetration
28					83	521	4	9	0.3	Overall
29					111	507	4	9	0.4	Penetration

The total diameter-to-distance ratio (DDR) of the karst cave group is the ratio of the diameter of all of the substitutive karst caves on the specified path and the length of the failure path.

The penetration failure mode refers to penetration along the cave group, and the other failure modes are regarded as overall failure.

Cave distance refers to the distance between the centroids of the karst caves.

Based on the comprehensive analysis of Path 2, 3 and 4, it can be seen that Path 3 has the advantage of tensile failure and Path 4 has the advantage of shear failure on the basis of maintaining the same DDR value, as shown in Fig. 9. Similar failure modes can be obtained by analyzing the failure cracks of single cave and cave groups on different paths. As can be seen from Table 3, after a simple analysis of the distribution of the karst cave group, this table allows for the quick identification of individual caves corresponding to the same failure pattern as the karst cave cluster in lieu of parameters, thus achieving a simplified treatment of the karst cave group.

## Stability analysis of a rock slope based on the upper bound method

In the previous section, based on the failure characteristics of a single cave with different shapes at different positions, the substitution schemes for cave groups to achieve similar failure paths were presented. The following will be a theoretical analysis of the penetration failure of a single cave between parallel structural planes is carried out.

### Derivation of slope stability analysis equation based on the upper bound method

The results of the numerical simulations reveal that when the cave penetration failure occurs, the angle between the failure path and the horizontal plane is generally in the range of 45°–90°, while a change in the failure angle of 15° results in an answer error of less than 1% when the upper limit method is used for analysis^38^. Therefore, for high-inclination rock slopes with karst caves, statistics and kinematics of the plastic limit analysis approach can be used to determine the upper bound of the true solution of the slope stability.

It is assumed that the rock mass is a rigid body that will not be damaged under external forces and gravity. Moreover, it is assumed that the displacement of adjacent structural planes is set to be continuous. Then, in the upper bound analysis, the internal energy represents the internal energy dissipation rate on the unstable rock mass and the velocity section. The power of external force includes the virtual power made by the equivalent load produced by the self-weight of the rock mass on the velocity field permitted by the motion, and the virtual power equation can be obtained by equating the dissipation rate of the internal energy of the system with the external power:

1 $$\dot{{W}_{int}}=\dot{{W}_{ext}}$$

**Figure 10:**
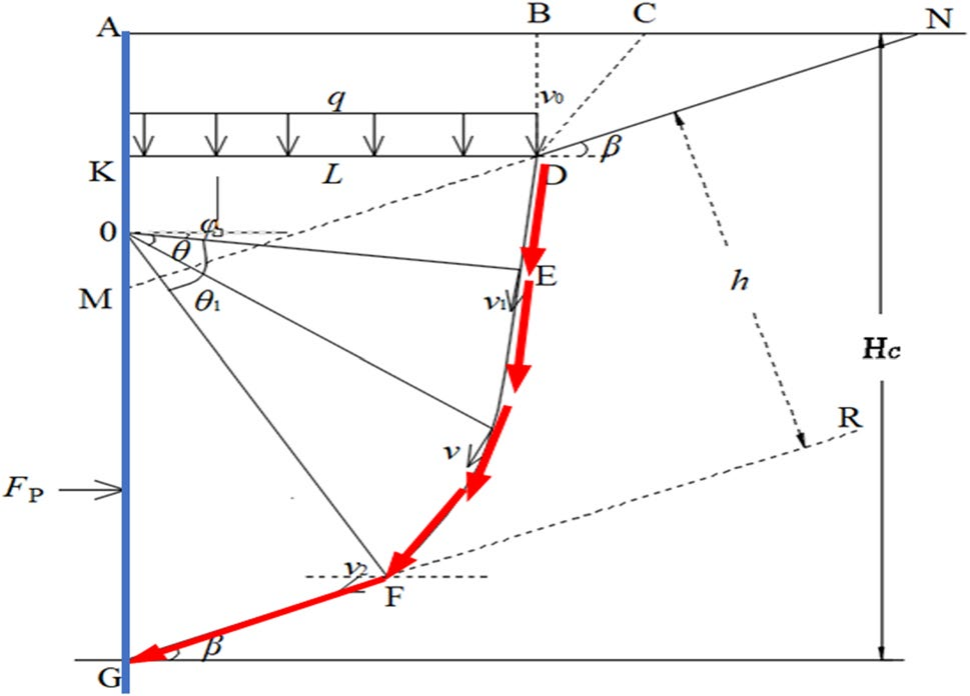
Upper bound analysis model for stability of rock slopes with structural plane and karst cave development.

As shown in Fig. 10, DE is a straight line, and EF is approximately a logarithmic spiral curve. OE is the starting point of the logarithmic spiral, and point D is the starting point of the rock bridge failure. DK is a constructed virtual working surface, which is subjected to the gravity of the upper rock mass ACDK, where the angle between BD and DC is 45° + φ/2. HC indicates the depth of the lower structural plane on the slope. The detailed derivation process can refer to the relevant papers of the author (Xu Jin, Yansen Wang. Stability analysis and support design methods for rock foundation pit with combination of structural plane and karst cave. *Advances in Civil Engineering*, vol. 2022, 13 pages, 2022.).

*c*_*1*_ and *φ*_*1*_ are the shear strength indices of the rock mass; and *c*_2_ and *φ*_2_ are the shear strength indices of the structural plane. The discounted strength parameter, with *K* as the safety factor of the foundation pit, is:

2 $$\mathrm{tan}{\varphi }_{m1}=\mathrm{tan}{\varphi }_{1}/K \quad{c}_{m1}={c}_{1}/K,$$

3 $${\mathrm{tan}{\varphi }_{m2}=\mathrm{tan}{\varphi }_{2}/K\quad c}_{m2}={c}_{2}/K.$$

The total work of the external forces is equal to the sum of the work done by the self-weight of the sliding rock mass, the earth pressure, the external force equivalent to the weight of the overlying rock, and the supporting force, as shown in formula (5):

4 $${W}_{total}={W}_{BCEO}+{W}_{OEF}+{W}_{q}+{W}_{OFG}+{W}_{P}.$$

The above equation can then be rewritten as

$$A{L}^{2}+BL+C{r}_{0}^{2}+D{r}_{0}+E{r}_{0}L+F=0,$$

where $$A=\mathrm{\gamma cos}{\varphi }_{m1}\cdot \mathrm{tan}\beta .$$

$$B=\mathrm{\gamma cos}{\varphi }_{m1}\frac{h}{\mathrm{cos}\beta }-{c}_{m1}\cdot \mathrm{cos}{\varphi }_{m1}\mathrm{tan}\beta +q\mathrm{cos}{\varphi }_{m1},$$

$$\left.C=\frac{1}{2}\gamma \left\{\frac{{T}^{3}\cdot [\left(3\mathrm{tan}{\varphi }_{m1}\cdot \mathrm{cos}({\varphi }_{m1}+{\theta }_{1}\right))+\mathrm{sin}{(\varphi }_{m1}+{\theta }_{1})]-4\mathrm{sin}{\varphi }_{m1}}{1+9{\mathrm{tan}}^{2}{\varphi }_{m1}}+\right.\left.{T}^{2}\cdot \mathrm{cos}{\varphi }_{m2}\cdot \mathrm{tan}\left(90-{\varphi }_{m1}-{\theta }_{1}\right)\right\}+\frac{1}{2}\gamma \cdot {\mathrm{cos}}^{2}{\varphi }_{m1}\cdot \mathrm{sin}{\varphi }_{m1}\right),$$

$$D=-\frac{{c}_{m1}\left({T}^{2}-1\right)}{\mathrm{tan}{\varphi }_{m1}}-{c}_{m2}\cdot {T}^{2}\mathrm{cos}{\varphi }_{m2}\cdot \mathrm{tan}\left(90-{\varphi }_{m1}-{\theta }_{1}\right),$$

$$E=-\gamma \mathrm{cos}{\varphi }_{m1}\cdot \frac{T}{\mathrm{cos}\left(90-{\varphi }_{m1}-{\theta }_{1}\right)},$$

$$F=-{c}_{m}\cdot \mathrm{cos}{\varphi }_{m1}\cdot \frac{h}{\mathrm{cos}\beta }+{F}_{p}\cdot T-\mathrm{cos}{\varphi }_{m1}\cdot \mathrm{sin}{\varphi }_{m1},$$

$$q=\left[{(N-L)}^{2}\frac{{sin}_{\beta }^{2}}{cos\beta }\cdot \frac{1}{2}\gamma +{\left(N-L\right)}^{2}\mathrm{tan}\beta \cdot \mathrm{cos}\beta \cdot \frac{1}{2}\gamma \mathrm{tan}{\varphi }_{m2}-{c}_{m2}(N-L)\mathrm{tan\beta }\cdot\upgamma \right]/L,$$

5 $$and\, N=\left[{H}_{c}-\frac{h}{\mathrm{sin}\left({90}^{0}-\beta \right)}\right]\cdot \mathrm{tan}\left({90}^{0}-\beta \right).$$

To find the position where the penetration of the karst cave between the structural planes occurs, that is, to obtain the minimum weight required for the overlying rock mass, a minimal value of L is required, such that $dL/d{r}_{0}=0$.

6 $$2C{r}_{0}+D+EL=0,$$

7 $$and\, L=f\left(\gamma ,{c}_{m1},{\varphi }_{m1},\beta ,{r}_{0},{c}_{m2},{\varphi }_{m2},{H}_{c}\right)$$

The safety factor when the cave undergoes penetration failure and the supporting force ${F}_{p}=0$ can be expressed as

8 $$K=f\left(\gamma ,{c}_{m1},{\varphi }_{m1},\beta ,L,{c}_{m2},{\varphi }_{m2},{H}_{c}\right)$$

When sliding of the rock mass occurs along the fracture surface, i.e., K < 1, the lateral pressure is

9 $${F}_{p}=-(A{L}^{2}+BL+C{{r}_{0}}^{2}+D{r}_{0}+E{r}_{0}L-{c}_{m1}\cdot \mathrm{cos}{\varphi }_{m1}\cdot \frac{h}{\mathrm{cos}\beta }-\mathrm{cos}{\varphi }_{m1}\cdot \mathrm{sin}{\varphi }_{m1})/\mathrm{T}.$$

In the presence of caves, the rock mass parameters (*c*_1,_ *φ*_1_) were discounted according to the ratio of the major axis (or diameter) of the cave to the length of the failure path. A change in the value of *φ*_1_ for the same rock mass has a small effect on the slope stability factor, so the focus was on the *c*_1_ value of the rock mass.

The above equations were programmed into MATLAB to obtain the solutions.

### Comparison between calculation and simulation results of the safety factor

**Figure 11:**
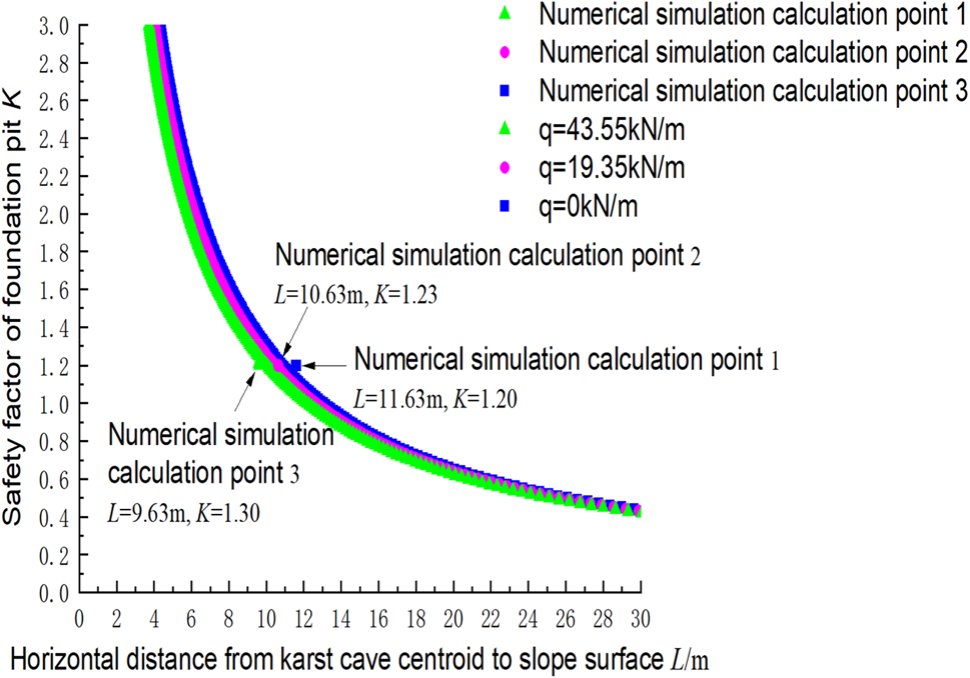
Comparison of theoretical and numerical results (60° inclination angle).

The parameters of the numerical simulation (c_1_ = 300 kPa, φ_1_ = 30°, c_2_ = 144 kPa, φ_2_ = 30°, γ = 23 kN/m^3^, and *H*_C_ = 25 m) were substituted into Formula (8), and the safety factors under inclination angles of 60° and 70° were calculated. Figure 11a,b shows the relationship between the safety factor and the position of the cave centroid. For example, at an inclination angle of 60° for the structural plane, the load of the overlying rock mass at point 3 obtained from the numerical simulation was 43.55 kN/m, *L* = 9.63 m. The theoretical safety factor K was 1.24 and the numerical simulation gave a safety factor of 1.3. The result of the theoretical calculation is slightly smaller than the result of the numerical simulation, which is on the conservative side. The calculation points are shown in Fig. 1.

### Influence of the presence of a structural plane on the stability of the foundation pit based on the upper bound method

**Figure 12:**
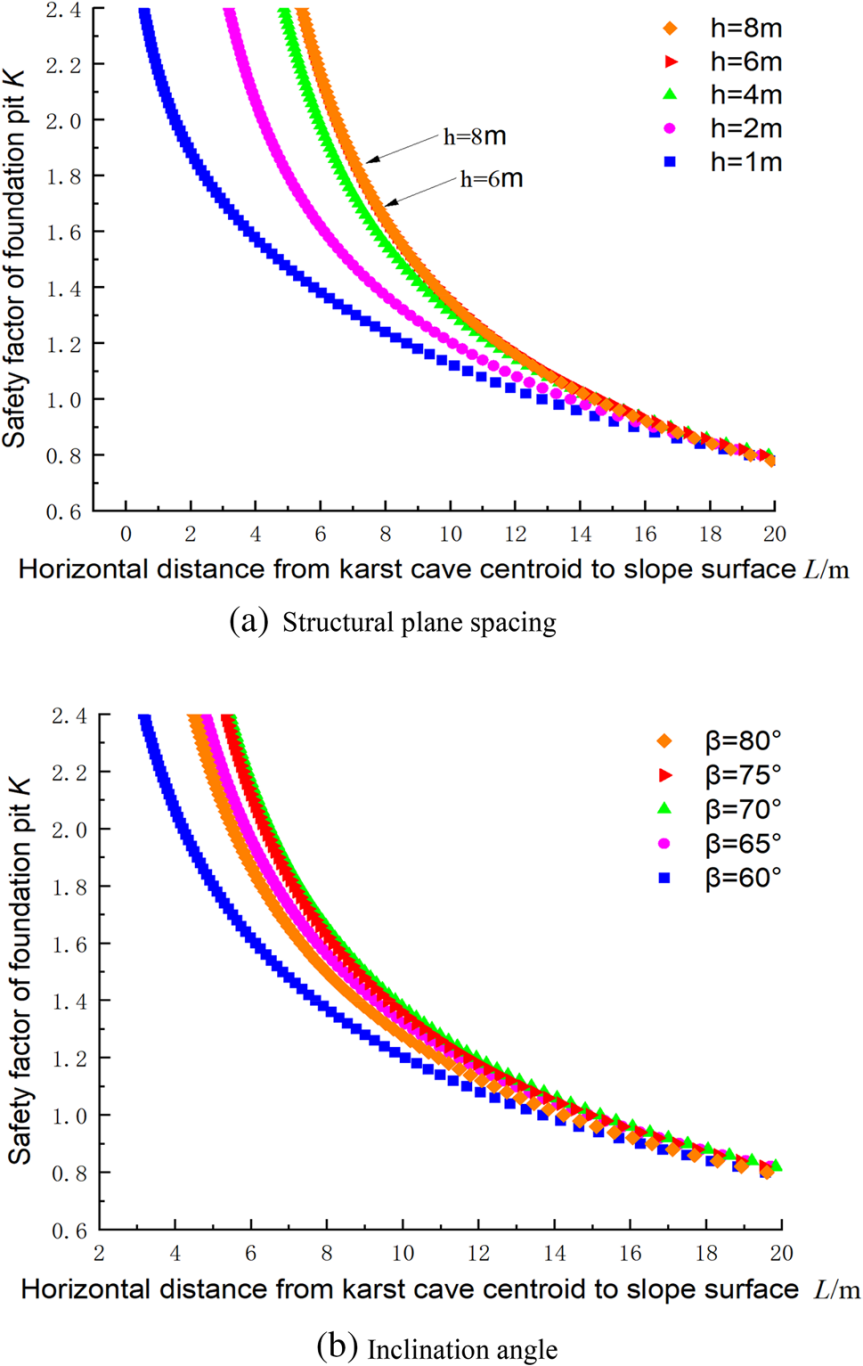
Influence of the structural plane occurrence on the safety factor.

Figure 12 shows the influence of the presence of a structural plane on the safety factor of the foundation pit, where the parameters c_1_ = 300 kPa, φ_1_ = 30°, c_2_ = 144 kPa, φ_2_ = 30°, γ = 23 kN/m^3^, and *H*_C_ = 25 m. Figure 12a, when the spacing of the structural planes was increased from 1 to 8 m, the safety factor of the caves at the same position increased and reached the highest value at 6 m. The results for the curves between 6 and 8 m were the same, indicating a positive correlation between the safety factor of the foundation pit and the spacing of the structural planes. When the maximum safety factor is achieved, the safety factor remains constant even if the spacing continues to increase.

Figure 12b shows the relationship between the safety factor and the position of the cave under different inclination angles of the structural plane. It can be seen that when the inclination angle was 60°, the safety factor was the smallest; and when the inclination angle was 70°, the safety factor reached the maximum value. And when the position of the cave is closer to the upper part of the pit, the deeper the depth of the lower structural plane, the smaller the influence of the inclination angle on the safety factor was, and the safety factor eventually tends to the same value.

### Influence of the rock mass parameters on the stability of the foundation pit based on the upper bound method

**Figure 13:**
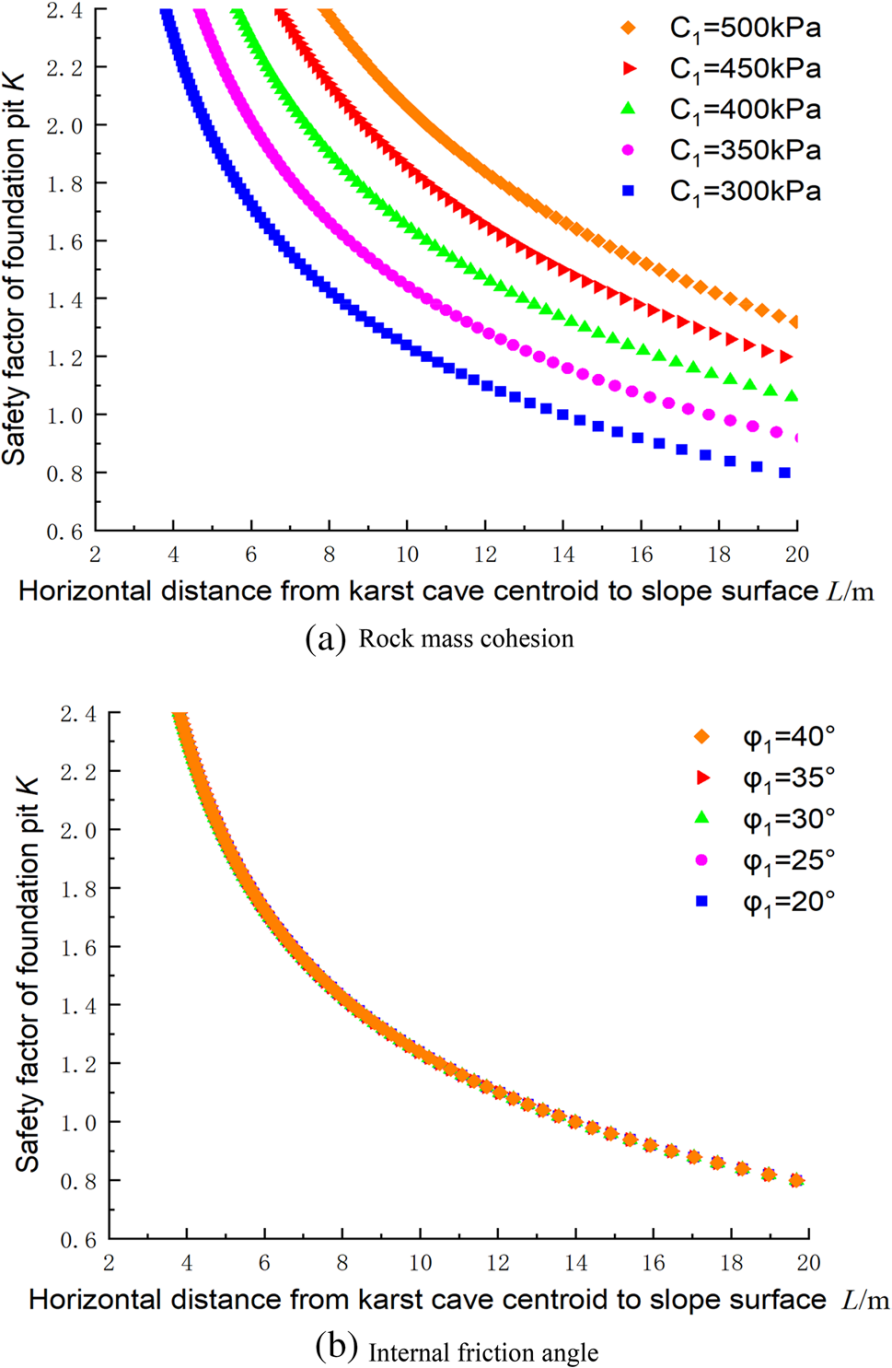
Influences of the rock mass parameters on the safety factor.

Figure 13a,b shows the influences of the mechanical parameters of the rock mass on the safety factor, where the parameters c_2_ = 144 kPa, φ_2_ = 30°, γ = 23 kN/m^3^, h = 2.25 m, *H*_C_ = 25 m, and β = 60°. As the cohesive force continued to decrease, i.e., the DDR increased, the influence of the cave at the same position on the safety factor of the foundation pit gradually increased, and the safety factor decreased. Moreover, the influence of the internal friction angle on the safety factor was relatively small, and the five curves almost overlap in the change interval from 20° to 40°, so the value of the internal friction angle does not play a decisive role in the influence of the safety factor of the foundation pit.

### Influence of the structural plane parameters on the stability the foundation pit based on the upper bound method

**Figure 14:**
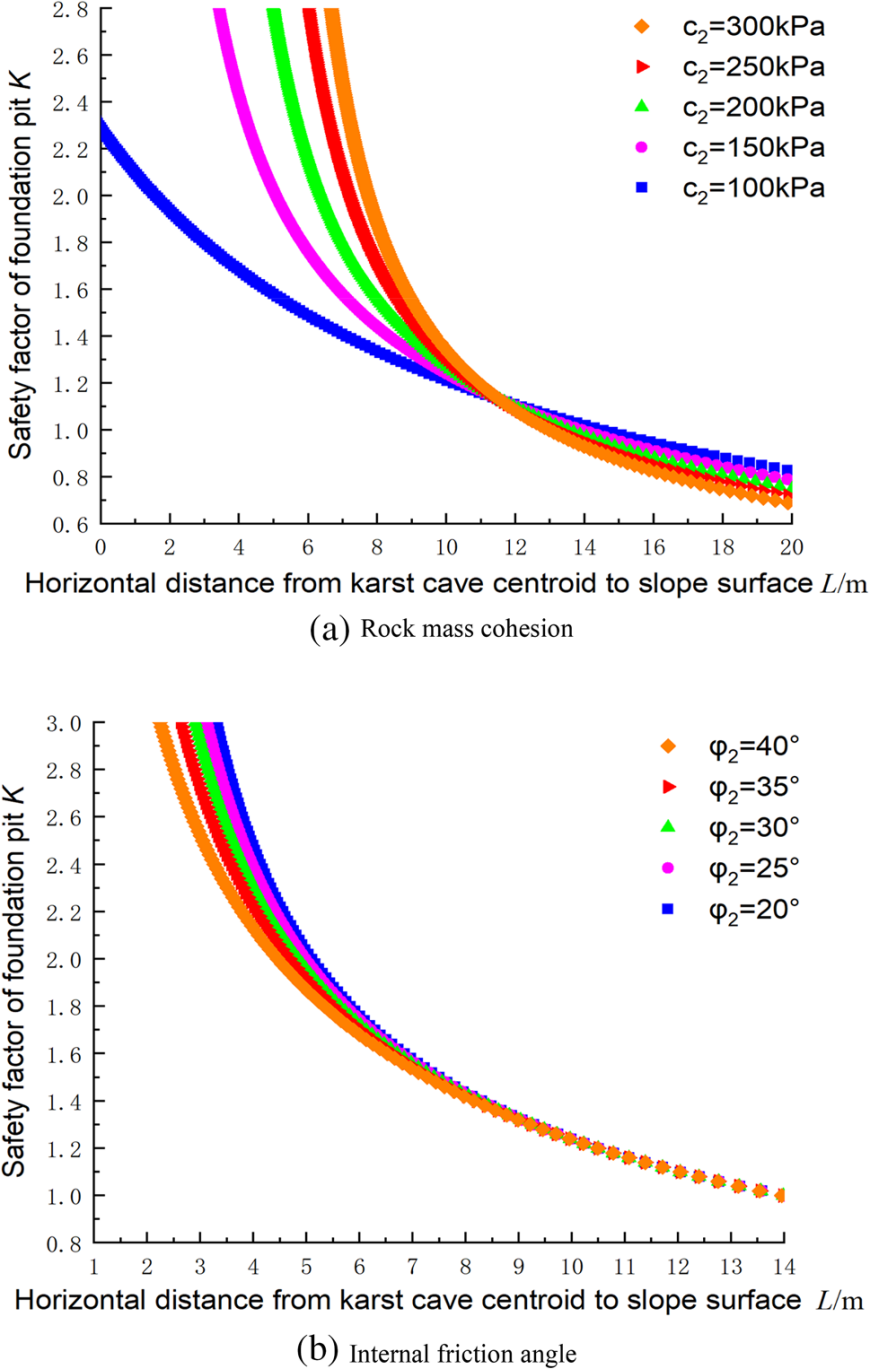
Influences of the structural plane parameters on the safety factor.

Figure 14a,b shows the influences of the mechanical parameters of the structural plane on the safety factor of the foundation pit, where the parameters c_1_ = 300 kPa, φ_1_ = 30°, γ = 23 kN/m^3^, *H*_C_ = 25 m, β = 60°, and h = 2.25 m. By changing the cohesive force of the structural plane, it was found that the five curves intersect at a distance of 11 m from the centroid. Thus, when the distance was less than 11 m, the lower the cohesion was, the lower the safety factor was. Beyond 11 m, the lower the cohesion, the higher the safety factor, but the magnitude of the increase was small. Similarly, by changing the internal friction angle of the structural plane, it was found that the influence on the safety factor was limited to a distance of 7 m from the cave centroid to the slope of the foundation pit. When the distance was > 7 m, the safety factor was not affected. Therefore, the friction angle only affected the penetration of the karst cave at the bottom of the foundation pit.

**Figure 15:**
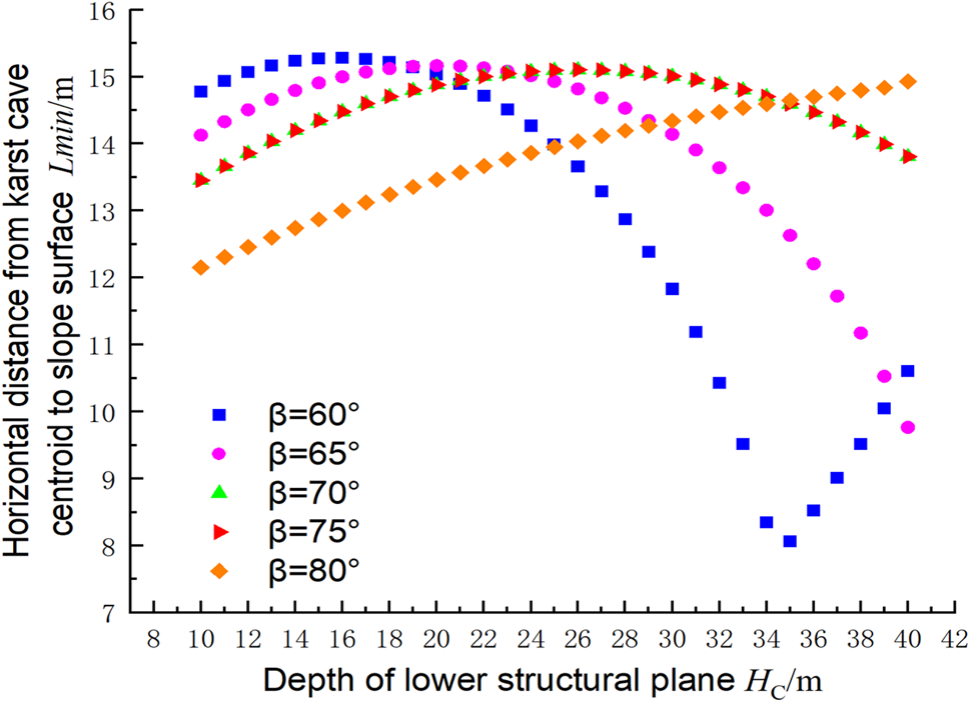
Relationship between the depth of the lower structural plane and the centroid position of the cave in the ultimate state.

For the same spacing of the structural planes, the relationship between the distance from the cave centroid to the slope of the foundation pit and the depth of the structural plane is shown in Fig. 15, where c_1_ = 300 kPa, φ_1_ = 30°, c_2_ = 144 kPa, φ_2_ = 30°, γ = 23 kN/m^3^, and h = 2 m. It can be seen from the figure that at the limit, when the structural plane’s inclination angle was 60°, the minimum distance decreased as the depth of the structural plane increased. At an inclination angle of 80°, the minimum distance increased as the depth of the structural plane increased. For the other angles between 60° and 80°, the minimum distance from the centroid to the slope surface initially increased and then decreased as the depth of the structural plane increased. The results therefore indicate that the risk of failure to the foundation pit is greater for slope angles of 60° than for the other angles.

### Relationship between the rock mass parameters and the cave position based on the upper bound method

**Figure 16:**
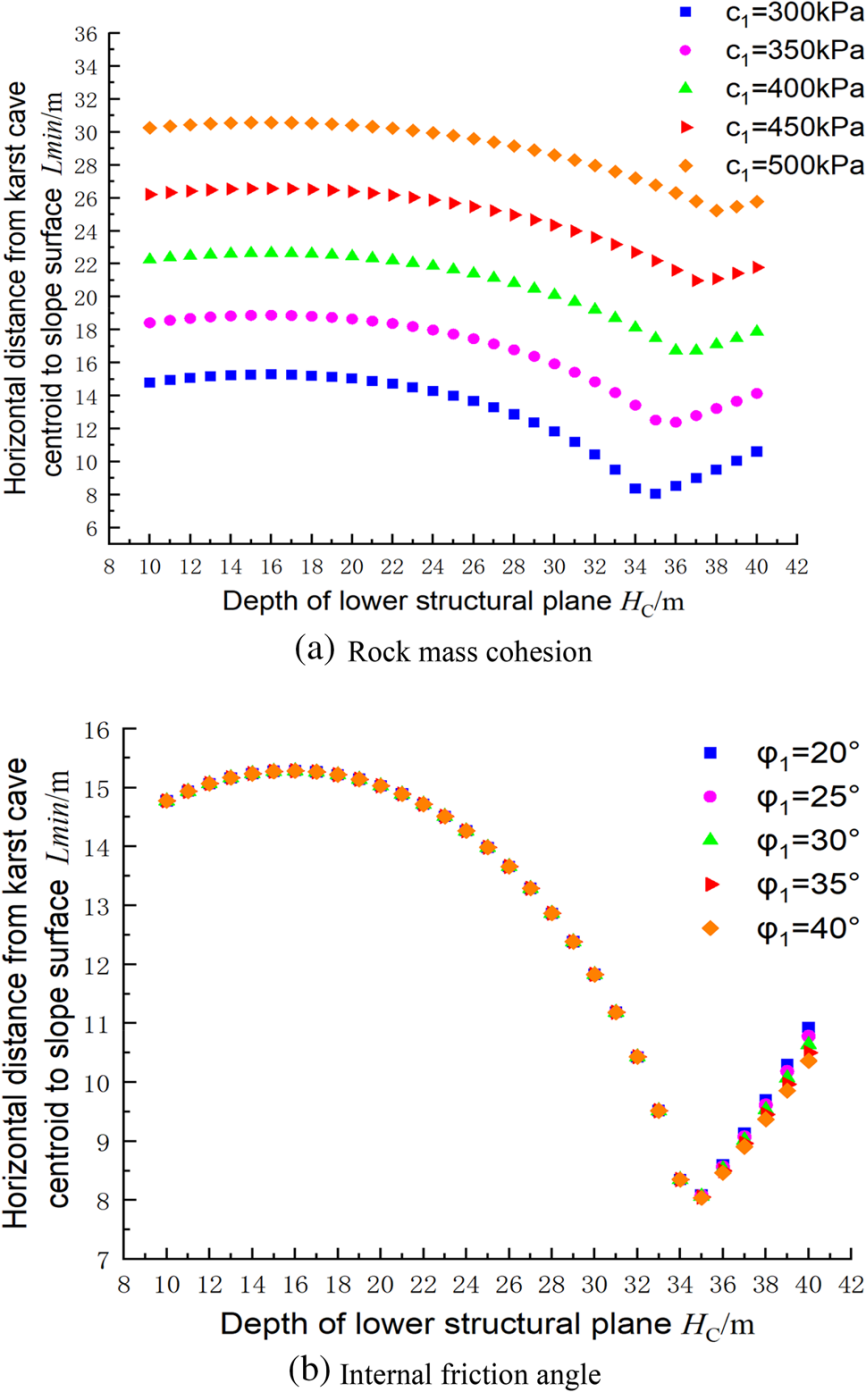
Influences of the rock mass parameters on the centroid position of the cave.

Figure 16 shows the influences of the rock mass parameters on the distance from the cave centroid to the slope surface. It can be seen that as the depth of the lower structural plane increases, the distance from the cave centroid to the foundation pit slope tends to increase slowly and then decrease gradually, reaching a minimum value before continuing to increase. In Fig. 16a, when c_1_ = 300 kPa, the minimum centroid distance occurs at a structural plane depth of about 34 m, and the minimum point gradually increases as the cohesion increases. In foundation pit engineering, the excavation depth is generally less than 34 m. Therefore, for urban foundation pit engineering projects, as the depth of the lower structural plane increases, the distance for the cave to reach the ultimate state gradually decreases. Changing the internal friction angle of the rock mass has little effect on the ultimate state of the foundation pit, and the safety factor of the foundation pit is primarily determined by the position of the cave relative to the structural plane in Fig. 16b.

**Figure 17:**
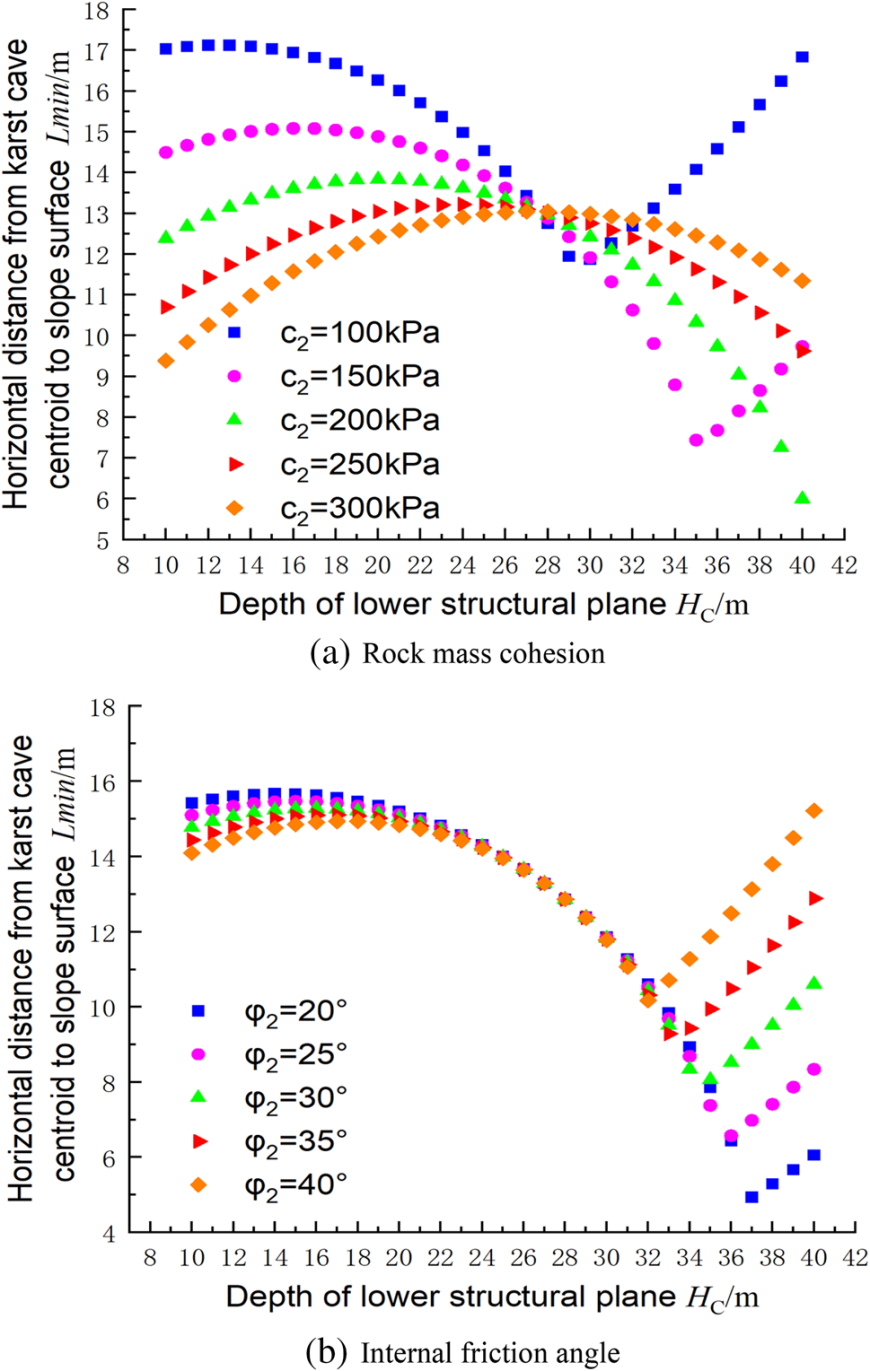
Influences of the structural plane parameters on the centroid position of the cave.

The influences of the mechanical parameters of the structural plane on the position of the cave centroid at the ultimate state is shown in Fig. 17. In Fig. 17a there is an intersection point, at a depth of approximately 28 m below the structural face, at which the position of the cave centroid at the ultimate state of the foundation pit does not change with the parameters of the structural plane. In Fig. 17a, there are two pivot points at c_2_ = 100 kPa and c_2_ = 150 kPa, beyond which the trend shows an inverse increase. When changing the angle of internal friction on the structural surface, as shown in Fig. 17b, there is a pivot point at each angle, with the turning point appearing deeper as the angle of internal friction increases.

### Supporting force calculation based on the upper bound method

**Figure 18:**
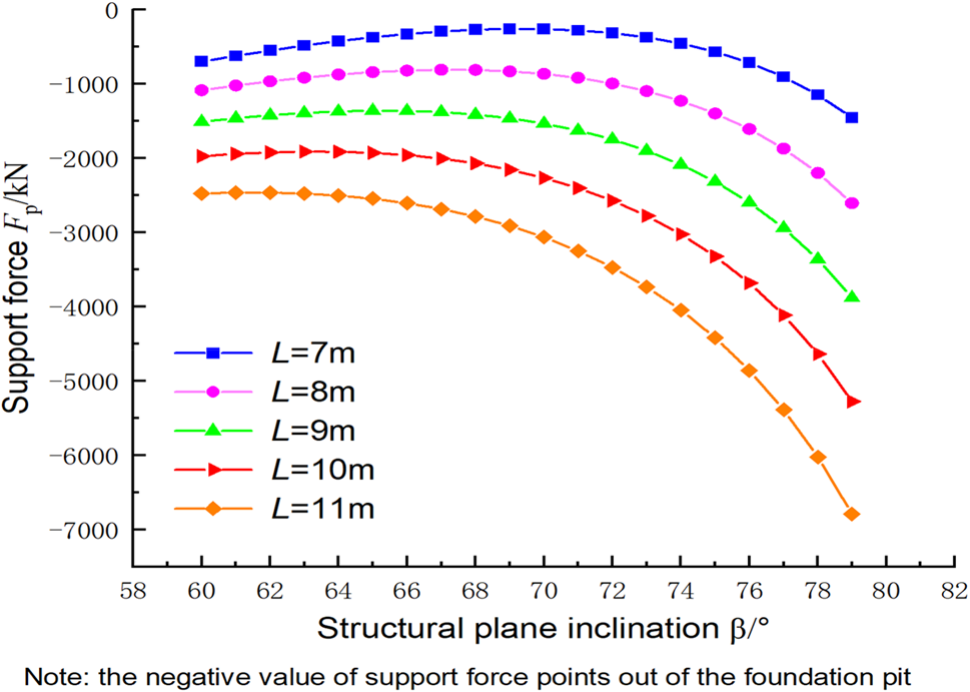
Relationship between the supporting force and the inclination angle of the structural plane.

**Figure 19:**
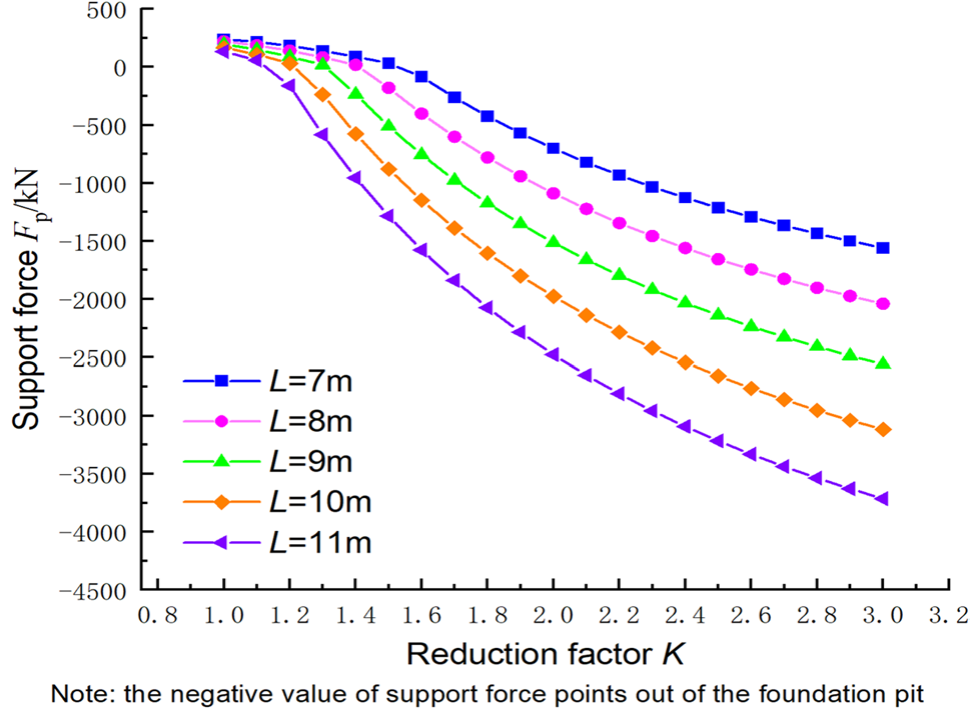
Relationship between the supporting force and the strength reduction coefficient.

In order to better guide the design of engineering support systems, the safety assessment equation needs to reflect the supporting force required by the foundation pit below a specified safety factor. Figure 18 reveals the relationship between the inclination angle of the structural plane and the supporting force (negative), where the parameters c_1_ = 300 kPa, φ_1_ = 30°, c_2_ = 144 kPa, φ_2_ = 30°, γ = 23 kN/m^3^, h = 2.25 m, and k = 2.0. When the inclination angle of the structural plane was changed from 60° to 80°, the supporting force required by the foundation pit exhibited an increasing–decreasing–increasing pattern. The maximum support force was required when the inclination angle reached 80°, and the distance from the cave centroid to the slope of the foundation pit was large. Therefore, the closer the cave between parallel structural planes is to the ground surface, the greater the support force required. The variations in strength coefficients of the support forces in Fig. 19 support this conclusion.

## Applicability of the different lateral pressure calculation methods for rock foundation pits

Table 4 lists the common methods for calculating the lateral soil pressure on the side of piles, using upright slopes and no overload at the top level of the slope. Compared to other methods, the Rankine’s lateral earth pressure formula (without considering the cohesion of the rock mass) obtained the smallest value. If the friction angle between the rock mass and the wall is taken into account, the results of the Coulomb’s lateral earth pressure formula at 10 m and 20 m are much greater than the results of the Rankine’s lateral earth pressure formula at the same calculation depths, but the results are similar at a depth of 30 m. For rock foundation pits, if there is no outwardly inclined structural plane within 10 m, the rock mass has a certain self-stabilizing ability. Therefore, Coulomb's formula for lateral earth pressure may overestimate the pressure in the shallow region.

**Table 4:** Earth pressure calculation table.

Basic calculation parameters								
Active earth pressure calculation methods	Soil weight (kN/m^3^)	Internal friction angle (°)	Cohesion (kPa)	Poisson's ratio	Additional load on slope top (kN/m^2^)	Angle between the fracture surface and the horizontal plane (°)	Internal friction angle of outwardly inclined structure plane (°)	Cohesion of outwardly inclined structure plane (kPa)
	23	45	150	0.17	0	60	15	0

Active earth pressure calculation methods			Calculated depth (m)		Active earth pressure resultant force (kN)		Comment	
Rankine’s active earth pressure			0		0		Cohesion not considered; failure angle: 45° + φ/2	
			10		197.3			
			20		789.2			
			30		1775.8			
Coulomb’s active earth pressure			0		0		Friction angle between rock mass and wall: 0.33φ; failure angle: 45° + φ/2	
			10		966.4			
			20		1573.7			
			30		1814.9			
Wedge algorithm			0		0		Friction angle between fill soil and rock mass: 18°; failure angle: 60°	
			10		499.3			
			20		1997.1			
			30		4493.6			
Slip along the outwardly inclined rigid structure plane			0		0		Failure angle: 60°	
			10		542.9			
			20		2171.7			
			30		4886.4			
Upper bound method			0		0		No karst cave; failure angle: 60°	
			10		314			
			20		1834.7			
			30		4351.2			

The wedge algorithm in Table 4 considers the friction angle between the interfaces, and gives slightly greater results than the algorithm for the Coulomb’s active earth pressure when the friction angle between the soil and the rock is 18°. If the calculated depth of each layer is taken as the starting point of the slip surface (the angle of fracture is taken as 60°) and the earth pressure formula for slip along an outwardly inclined rigid structure surface is used, the earth pressure of each layer is increased higher than the result of the wedge algorithm. This is because the friction angle of the contact surfaces in the wedge algorithm uses an empirical parameter, whereas the value used in this study was the minimum value within the empirical parameter range. In the equation for calculating the slip along an outwardly inclined rigid structural surface, the shear strength value measured via indoor shear tests is used and the number of parameters for the slip fracture surface calculation are different between the two.

If the position of the karst cave is in the top of the pile, Formula (9) based on the upper bound method degrades to a cave-free condition. When karst caves are not taken into account, the result of the upper bound method is slightly lower than that of the earth pressure of the slip along the outwardly inclined rigid structural surface. This is because the result of the upper bound method is the optimal solution that satisfies the energy equation, that is, the minimum residual thrust when the slope reaches the ultimate state. In a sense, the result calculated along the outward sloping structural plane satisfies the requirements of the upper bound method. However, for the wedge algorithm, an accurate value of the interface friction angle is required in order to obtain accurate calculation results.

## Engineering case validation taking Xuzhou Metro Sanhuan South Road Station as an example

**Figure 20:**
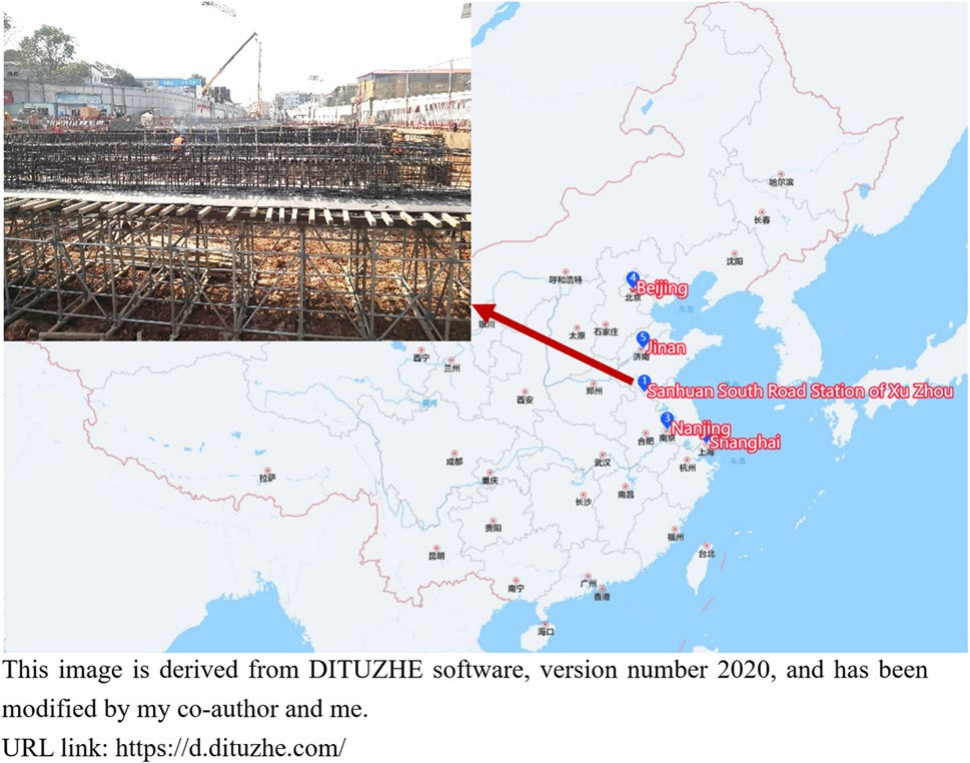
Location map of Sanhuan South Road Station.

Xuzhou Metro Line 3 is the main line of Xuzhou Rail Transit in the North–South direction, of which Sanhuan South Road Station is the transfer station for planned Line 4 as shown in Fig. 20 (Fig. 20 shows a portion of the area from DITUZHE software by marking key location points.). The maximum excavation depth is about 30 m, and the west side of the foundation pit is the slope of the bedding structural plane with the inclination angle of 70°. The enclosure structure mainly adopts Ø 1000 mm@1500 mm bored piles. The first support in the foundation pit is poured with C35 concrete, the second support is steel support (diameter 800 mm), and the third steel support is set in local areas.

**Figure 21:**
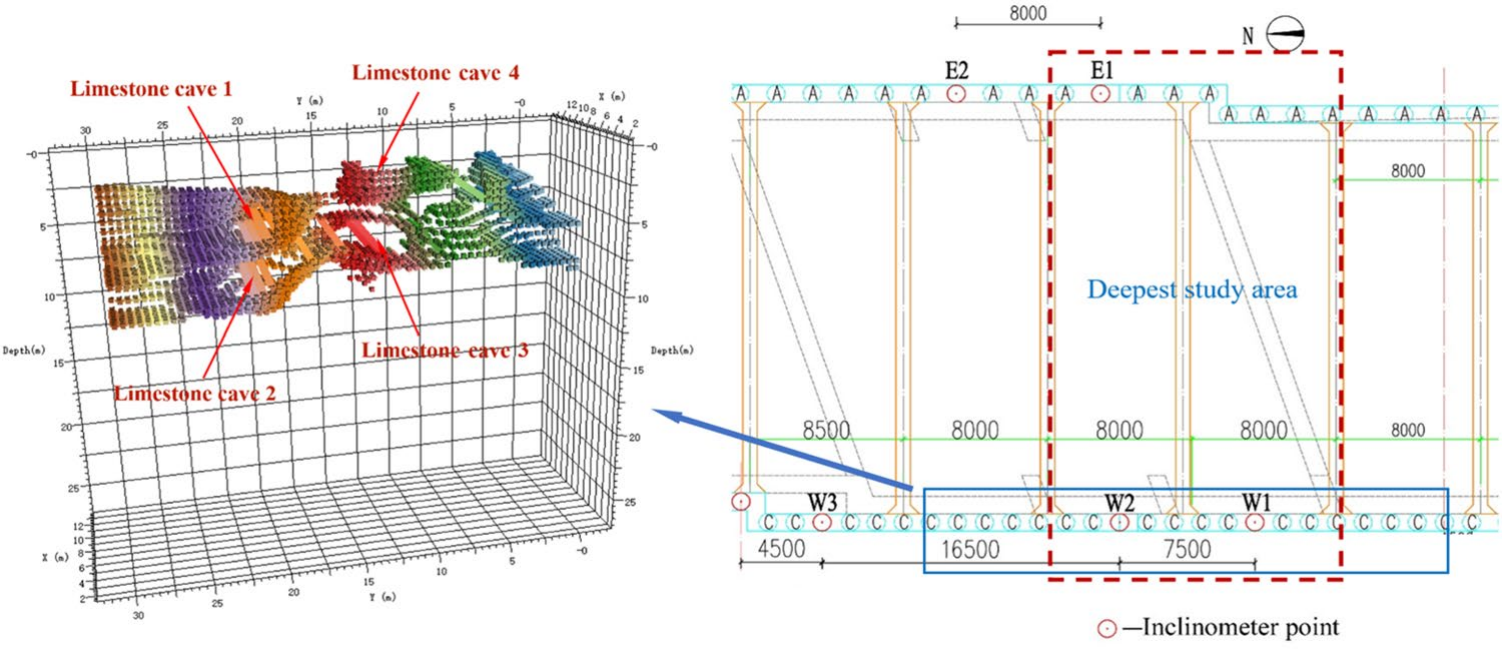
Distribution of karst caves in foundation pit.

Sanhuan South Road Station is adjacent to Mount Zhai. Local surface water infiltrates from the top of the mountain and intrudes into the lower rock mass, which is easy to form a large number of karst caves. According to field pumping test, the permeability coefficient of rock mass in the site is about 3.5 m/day. Figure 21 is the distribution map of partial karst caves on the west side of foundation pit of Sanhuan South Road Station by seismic tomography technology adopted by Professor Song, a member of our scientific research team.

In this paper, W1 retaining pile in the area of maximum excavation depth is selected for analysis as shown in Fig. 21. The analysis idea is to compare the horizontal displacement of the pile body monitored on site with the theoretical displacement of the pile body calculated by the elastic fulcrum method based on the upper limit method so as to verify the reliability of the calculation method. The karst cave is transformed by approximation, i.e. the contour of complex karst cave is transformed into approximate shape with equal DDR value for theoretical analysis.

**Figure 22:**
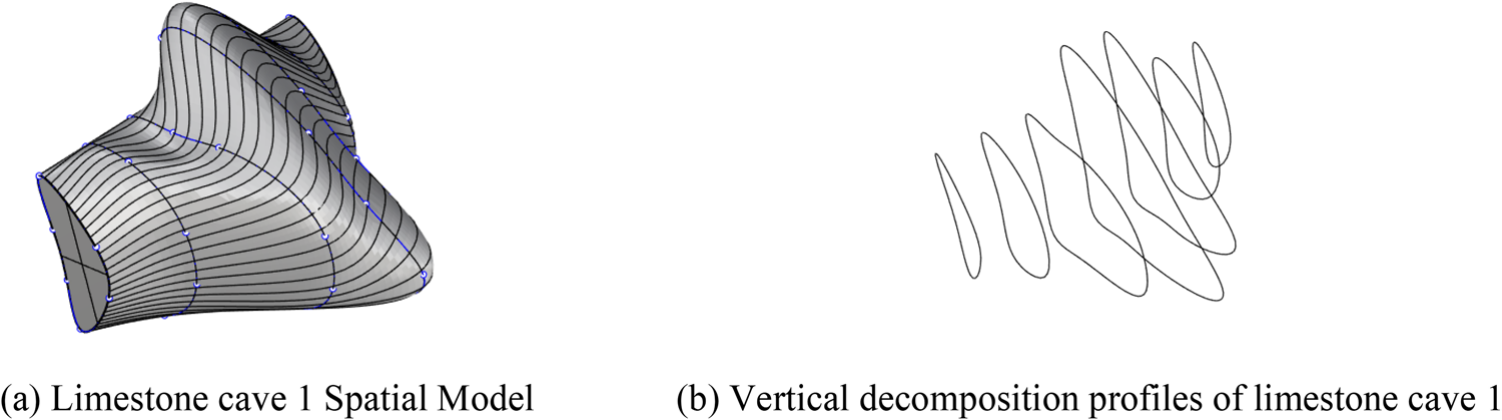
Resolution schematic diagram of cave contour based on wave velocity dot matrix of seismic tomography.

Taking the contour of cave 1 in Fig. 21 as an example, the cave was simplified to a regular shape with the DDR value of the original cave unchanged, and both were simulated by uniaxial compression and uniaxial shear test with PFC2d software, as shown in Fig. 22a,b, to prove that the simplified cave shape was similar to the original cave.

**Figure 23:**
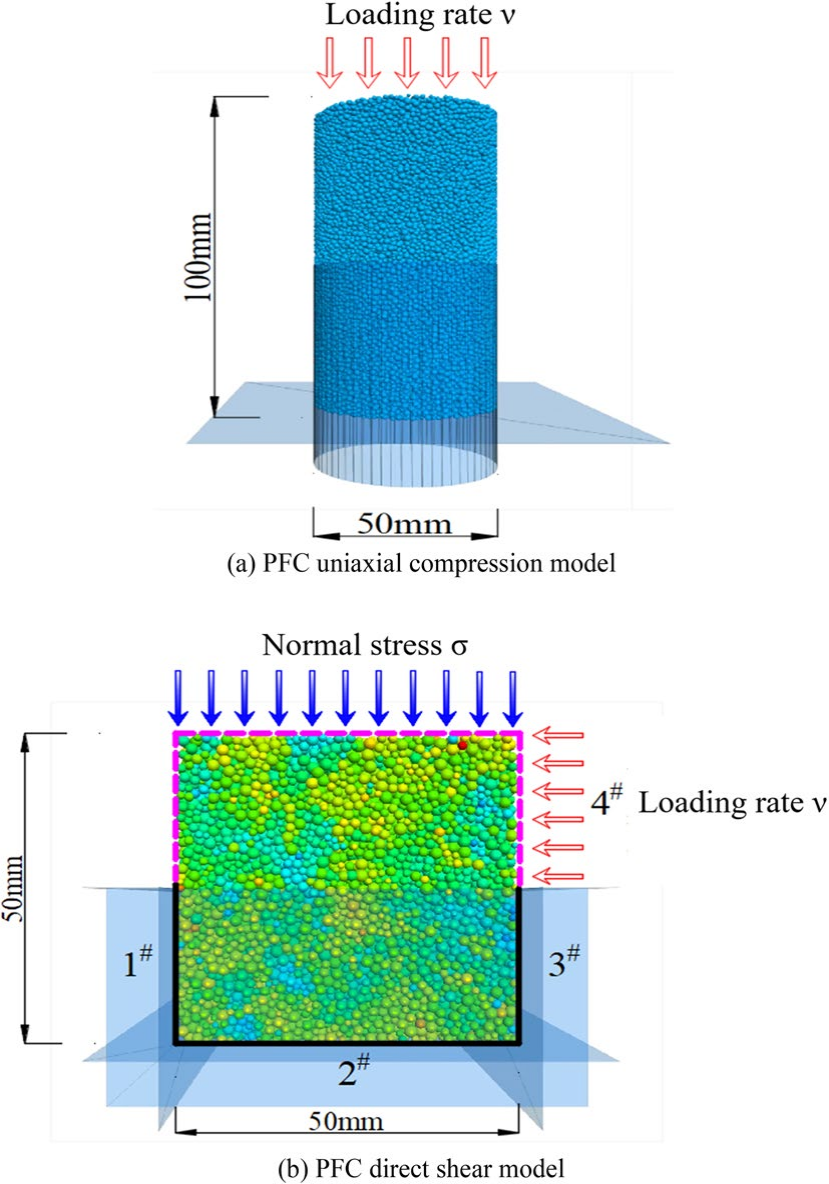
PFC simulation diagram.

As shown in Fig. 23a, the karst cave was equiproportionally reduced and placed inside a cylindrical rock mass with a width of 50 mm and a height of 100 mm, and loaded vertically at a speed of 0.002 m/s. The direct shear analysis uses the same method to place the karst cave inside a cubic rock mass with a length of 50 mm and load it horizontally at a speed of 0.01 m/s as shown in Fig. 23b. The parameters of the rock body at the time of study were taken as limestone as shown in Table 5.

**Table 5:** Basic microscopic parameters of materials.

Particle density (ρ)/g/cm^3^	Contact modulus (*E*_c_)/GPa	Friction coefficient	Stiffness ratio (K)	Parallel bonding modulus $\stackrel{-}{(Ec})$/GPa
2.5	1.4	0.8	2.0	2.4

**Figure 24:**
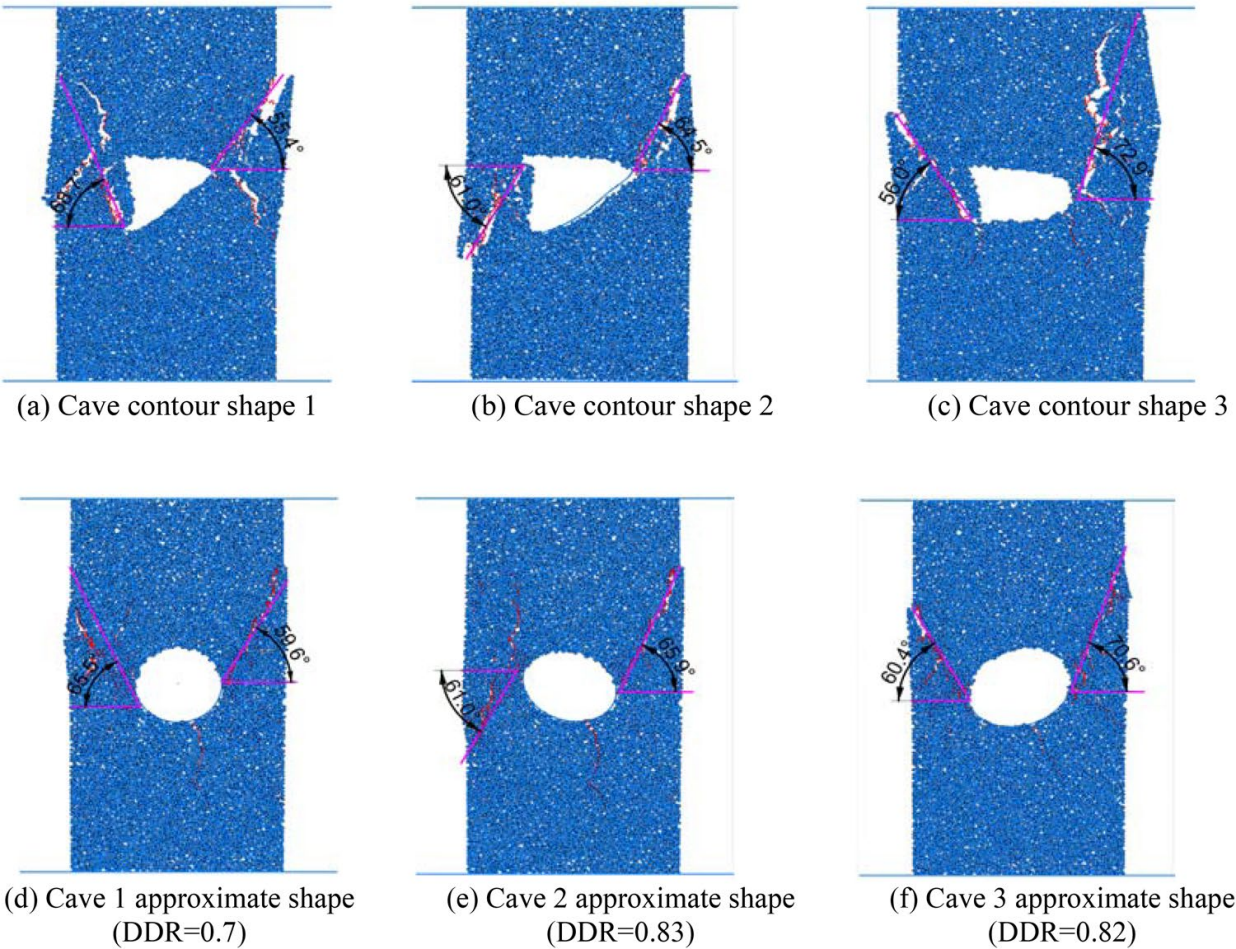
Uniaxial compression failure nephograms of each section profile of simplified caves and original caves.

**Figure 25:**
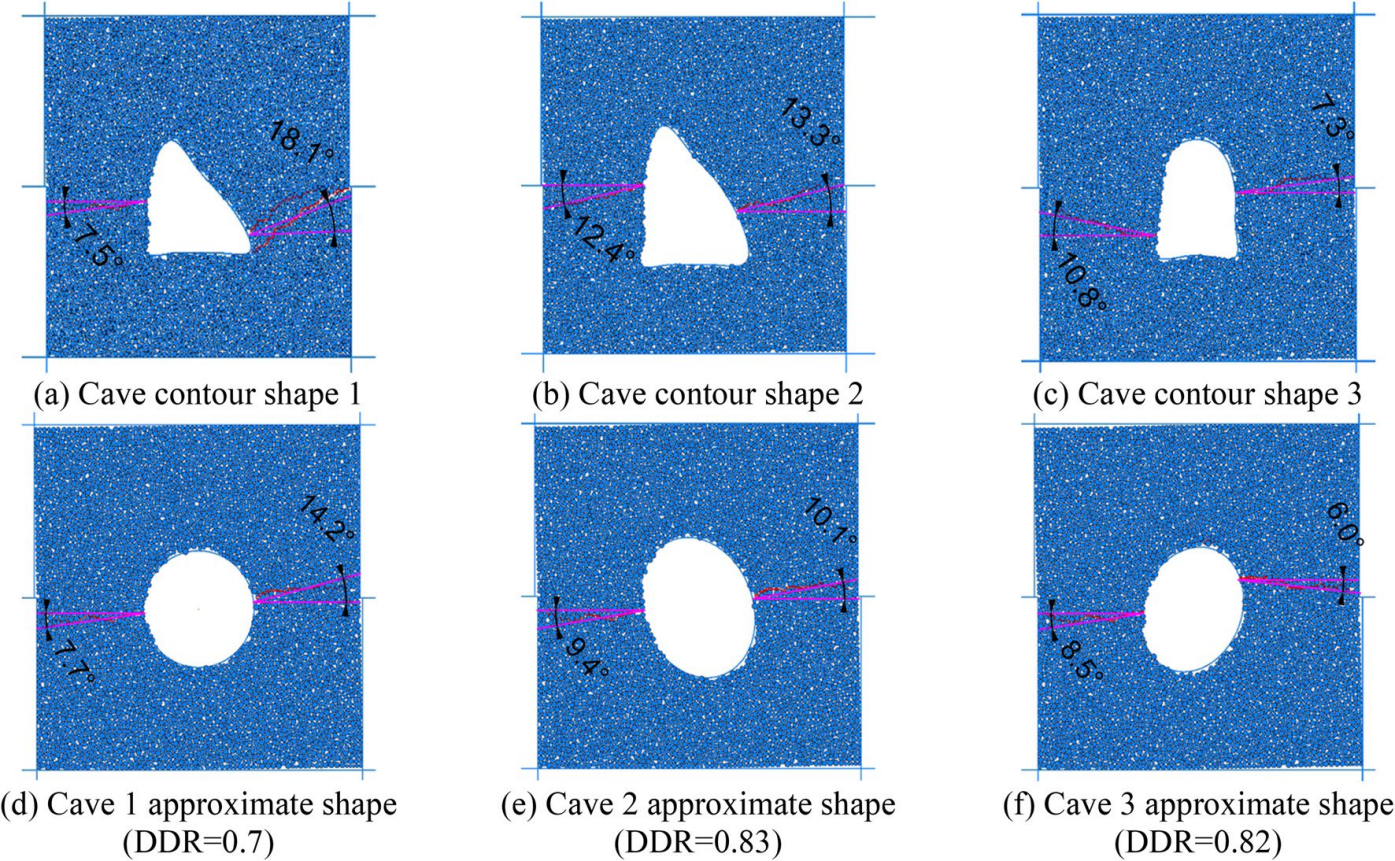
Direct shear failure nephograms of each section contour of simplified caves and original caves.

Select three contour figures with large DDR value in Fig. 22b for stress analysis. Figure 24a–c show the fracture formed by two-dimensional uniaxial compression of the original karst cave contour, and Fig. 24d–f show the fracture formed by compression of the corresponding approximate regular shape. According to the included angle between the fracture and the horizontal line, it can be found that the difference between the original karst caves and the regular karst caves is within 5°. PFC direct shear test is carried out in the same way, as shown in Fig. 25a–f. The results also show that the fracture angle error of the above two karst caves is concentrated within 5°. The fracture angle error within 15° has little effect on the analysis results of the upper bound analysis method, so it can be proved that the failure effect of the original karst cave shape is similar to that of the regular shape.

**Figure 26:**
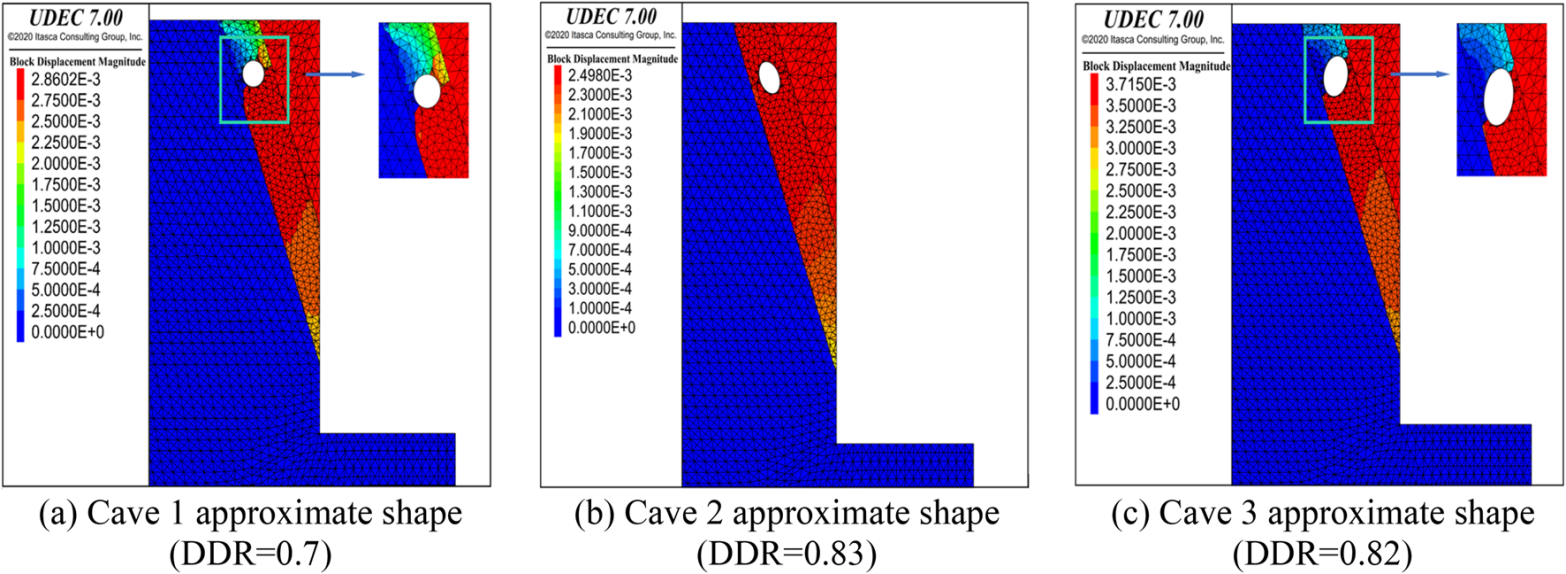
Failure displacement nephogram of each karst cave contour under plane strain model.

**Figure 27:**
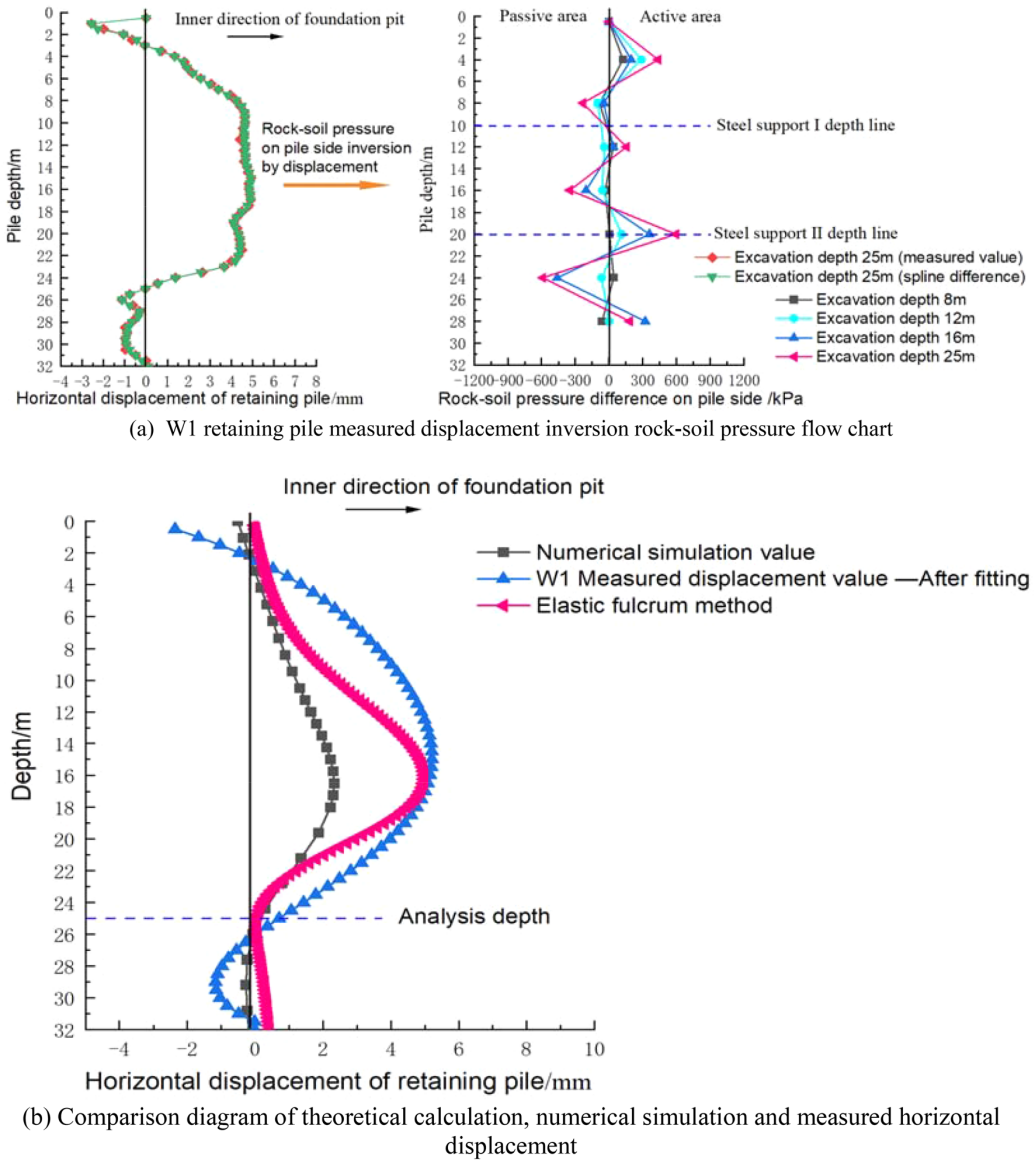
Theoretical analysis process diagram of W1 retaining pile.

The plane contours of the above three karst caves were brought into UDEC numerical software for stability analysis. The parameters of rock mass and structural plane are shown in Table 6. As shown in Fig. 26a–c, karst cave contour 1 and karst cave contour 3 can produce broken line failure of rock mass, while the included angle between the long axis of karst cave contour 2 and the horizontal line is between 40° and 80° and cannot form penetration. The above conclusions are the same as those in chapter 2 of this paper. Figure 27a shows the measured horizontal displacement curve of W1 excavated to a depth of 25 m and the distribution curve of rock-soil pressure difference at the pile side after inversion through cubic spline interpolation, so as to calculate the horizontal reaction coefficient as k = 20 MN/m^3^.

**Table 6:** Numerical calculation parameters.

Layer	Geotechnical layer type	Specific weight (kN/m^3^)	Cohesion (kPa)	Internal friction angle (°)
1	Structural plane	19.5	150.0	30.00
2	Limestone (moderately weathered rock)	26.8	300.0	45.00

When the safety factor *K* is taken as 1.0, the residual sliding force of broken line sliding when the excavation depth is 25 m is calculated by taking the centroid position of karst cave *L* = 3.94 m into Formula (9), and the residual earth pressure is brought into the elastic fulcrum method model with triangular distribution to calculate the horizontal displacement. Figure 27b shows the comparison of horizontal displacement between numerical simulation, elastic fulcrum method and field measurement. It can be seen that the deformation trend of the elastic fulcrum method is similar to the measured displacement value, while the calculation result of the numerical simulation method is slightly smaller. Therefore, it can be proved that the calculation result of rock-soil pressure calculated based on the upper bound method is acceptable.

## Conclusion

In summary, the equation for calculating the stability of pit slopes based on the upper limit analysis can meet the calculation requirements for conditions with and without karst caves, and the theoretical analysis of complex cave conditions can be further simplified with the help of the karst cave group substitution table listed in Table 4 to achieve the theoretical analysis of complex rock slopes. At the same time, based on the above research, the following conclusions can be drawn:

1. When the dip angle of structural plane is between 60° and 80°, there are three basic modes of slope failure caused by circular karst cave. The first one, slip body slides along upper structural plane at the shallow part of foundation pit, and the whole slides along lower structural plane after the crack penetrates the karst cave towards the outer side of foundation pit; The second, integral sliding of rock mass after straight penetration of cracks along single structural plane; The third, the sliding body slides along the lower structural plane near the free surface of the foundation pit. After the crack penetrates the karst cave towards the inner side of the foundation pit, the whole slides along the upper structural plane.
2. On the basis of maintaining the minimum distance diameter ratio, if the diameter of the cave on the 45° + φ/2 path is greater than that of the vertical cave, the 45° + φ/2path will become the dominant failure path; When the road strength is lower than 45° + φ/2, changing the diameter of karst cave can not improve the advantage.
3. The residual sliding force calculated based on the upper bound analysis principle is the minimum additional support force required for the rock mass to meet the limit equilibrium. Without considering the karst cave, the result is slightly smaller than the traditional calculation formula of earth pressure.
4. The complex geological analysis can be simplified through the replacement table of karst cave group and single karst cave, and then the rock slope can be analyzed quickly with the help of the stability calculation formula based on the upper bound principle, so as to provide guidance for engineering design.
5. In this paper, the cut surface of the karst cave is analyzed one by one, i.e. the longitudinal length of the karst cave is not considered. Therefore, the determination of the damage range of the spatial wedge can be based on the contour spacing of the minimum penetration failure DDR value and the spacing of the vertical structural plane.

## Data Availability

The data used to support the findings of this study are available from the corresponding author upon request.

## References

[1] Wu, S. G. *Stability and Control of Architectural Rock Slopes*. Dissertation, Chongqing University (2005).

[2] Kumsar H, Aydan O, Ulusay R (2000). Dynamic and static stability assessment of rock slopes against wedge failures. Rock Mech. Rock Eng..

[3] Moawwez MA, Wang J, Hussain MA (2021). Development of empirical correlations for limit equilibrium methods of slope stability analysis. Arab. J. Geosci..

[4] Sriyati R, Martini M, Mastura L, Shafira YA (2021). Investigation of potential landslides due to liquefaction on the balaroa road section using the limit equilibrium method. J. Appl. Eng. Sci..

[5] Azmoon B, Biniyaz A, Liu ZL (2021). Evaluation of deep learning against conventional limit equilibrium methods for slope stability analysis. Appl. Sci..

[6] Kim J, Salgado R, Lee J (2002). Stability analysis of complex soil slopes using limit analysis. J. Geotechnol. Geoenviron..

[7] Totonchi A, Askari F, Farzaneh O (2012). 3D stability analysis of concave slopes in plan view using linear finite element and lower bound method. Ijst-T Civ. Eng..

[8] Li X, He S, Wu Y (2012). Limit analysis of the stability of slopes reinforced with anchors. Int. J. Numer. Anal. Met..

[9] Wang L, Sun D, Li L (2019). Three-dimensional stability of compound slope using limit analysis method. Can. Geotech. J..

[10] Wang L, Zhao G, He F (2013). Analysis of slope stability containing weak layers. Adv. Civ. Eng. II PTS 1–4.

[11] Xie X, Liu Y, Han J, Xing L (2014). 3D finite element numerical analysis for stability calculation of high steep rock slope. Adv. Civ. Ind. Eng. IV..

[12] Dadashzadeh N, Duzgun HSB, Yesiloglu-Gultekin N (2017). Reliability-based stability analysis of rock slopes using numerical analysis and response surface method. Rock Mech. Rock Eng..

[13] Alqadhi S, Mallick J, Talukdar S, Bindajam AA, Saha TK, Ahmed M (2021). Combining logistic regression-based hybrid optimized machine learning algorithms with sensitivity analysis to achieve robust landslide susceptibility mapping. Geocarto Int..

[14] Ye J, Song J, Du S, Yong R (2021). Weighted aggregation operators of fuzzy credibility numbers and their decision-making approach for slope design schemes. Comput. Appl. Math..

[15] Wang Z, Lin M (2021). Finite element analysis method of slope stability based on fuzzy statistics. Earth Sci. Res. J..

[16] Pan J, Ma Y (2013). Stability analysis of an earth slope based on Taylor Series Method. Front. Green Build. Mater. Civ. Eng. III PTS.

[17] Song L, Yu X, Xu B, Pang R, Zhang Z (2021). 3D slope reliability analysis based on the intelligent response surface methodology. B Eng. Geol. Environ..

[18] Zhang T, Zeng P, Jimenez R, Li T, Feng X, Sun X (2022). System reliability analysis of soil slopes using shear strength reduction and active-learning surrogate models. Arab. J. Geosci..

[19] Zhang K, Cao P, Ma G, Fan W, Meng J, Li K (2016). A new methodology for open pit slope design in Karst–Prone ground conditions based on integrated Stochastic-Limit equilibrium analysis. Rock Mech. Rock Eng..

[20] Andriani GF, Loiotine L (2020). Multidisciplinary approach for assessment of the factors affecting geohazard in karst valley: The case study of Gravina di Petruscio(Apulia, South Italy). Environ. Earth Sci..

[21] Cui F, Li B, Xiong C, Yang Z, Peng J, Li J (2022). Dynamic triggering mechanism of the Pusa mining-induced landslide in Nayong County, Guizhou Province, China. Geomater. Nat. Hazard Risk..

[22] Sun B (2020). A computational method for two/three dimensional competitive fracture process simulation of rock-like materials due to cracking. Arab. J. Geosci..

[23] Huang Y, Yang S, Tian W, Zeng W, Yu L (2016). An experimental study on fracture mechanical behavior of rock-like materials containing two unparallel fissures under uniaxial compression. Acta Mech. Sin. Prc..

[24] Zhou XP, Bi J, Qian QH (2015). Numerical simulation of crack growth and coalescence in Rock-Like materials containing multiple pre-existing flaws. Rock Mech. Rock Eng..

[25] Park, N., Park, P., Hong, C., Jeon, S. Crack propagation and coalescence under uniaxial loading. In *Rock Mechanics: A Challenge For Society*. *ISRM Reg EUROCK Symposium on Rock Mechanics* (eds. Sarkka, P. & Eloranta, P.) 271–276 (2001).

[26] Yang SQ (2013). Study of strength failure and crack coalescence behavior of sandstone containing three pre-existing fissures. Rock Soil Mech..

[27] Huang D, Zhang XJ, Gu DM (2018). Failure pattern and evolution mechanism of locking section in rock slope with three-section landslide mode. Chin. J. Geotech. Eng..

[28] Chen GQ, Chen Y, Sun X (2020). Crack coalescence and brittle failure characteristics of open rock bridges. Chin. J. Geotech. Eng..

[29] Shen J, Karakus M (2014). Three-dimensional numerical analysis for rock slope stability using shear strength reduction method. Can. Geotech. J..

[30] Umrao RK, Singh R, Sharma LK, Singh TN (2017). Soil slope instability along a strategic road corridor in Meghalaya, north-eastern India. Arab. J. Geosci..

[31] Li H, Du H, Bai R, Liu G, Zhao M, Liu R (2021). The failure mechanism and stability of the end slope of inclined composite coal seam. Math. Probl. Eng..

[32] Lin H, Xiong W, Cao P (2013). Stability of soil nailed slope using strength reduction method. Eur. J. Environ. Civ. Eng..

[33] Rai R, Khandelwal M, Jaiswal A (2012). Application of geogrids in waste dump stability: A numerical modeling approach. Environ. Earth Sci..

[34] Dong M, Kulatilake PHSW, Zhang F (2018). Deformation and stability investigations in 3-D of an excavated rock slope in a hydroelectric power station in China. Comput. Geotech..

[35] Dong M, Zhang F, Lv J, Hu M, Li Z (2020). Study on deformation and failure law of soft-hard rock interbedding toppling slope base on similar test. B Eng. Geol. Environ..

[36] Marcato G, Mantovani M, Pasuto A, Zabuski L, Borgatti L (2012). Monitoring, numerical modelling and hazard mitigation of the Moscardo landslide (Eastern Italian Alps). Eng. Geol..

[37] Groneng G, Lu M, Nilsen B, Jenssen AK (2010). Modelling of time-dependent behavior of the basal sliding surface of the Aknes rockslide area in western Norway. Eng. Geol..

[38] You ZJ (2017). Geotechnical Plasticity Theory and Its Application in Underground Engineering.

